# Lineages of embryonic stem cells show non-Markovian state transitions

**DOI:** 10.1016/j.isci.2021.102879

**Published:** 2021-07-17

**Authors:** Tee Udomlumleart, Sofia Hu, Salil Garg

**Affiliations:** 1 Koch Institute for Integrative Cancer Research, Massachusetts Institute of Technology, Cambridge, MA 02142, USA; 2 Department of Biology, Massachusetts Institute of Technology, Cambridge, MA 02142, USA; 3 Harvard-MIT MD PhD Program, Harvard Medical School, Boston, MA 02115, USA; 4 Department of Pathology, Massachusetts General Hospital, Boston, MA 02114, USA

**Keywords:** Cell biology, Stem cells research, Developmental biology, Embryology, Systems and computational biology

## Abstract

Pluripotent embryonic stem cells (ESCs) constitute the cell types of the adult vertebrate through a series of developmental state transitions. These states can be defined by expression levels of marker genes, such as Nanog and Sox2. In culture, ESCs reversibly transition between states. However, whether ESCs retain memory of their previous states or transition in a memoryless (Markovian) process remains relatively unknown. Here, we show some highly dynamic lineages of ESCs do not exhibit the Markovian property: their previous states and kin relations influence future choices. Unexpectedly, the distribution of lineages across their composition between states is constant over time, contrasting with the predictions of a Markov model. Additionally, highly dynamic ESC lineages show skewed cell fate distributions after retinoic acid differentiation. Together, these data suggest ESC lineage is an important variable governing future cell states, with implications for stem cell function and development.

## Highlights

• Dynamics of embryonic stem cells switching between Nanog and Sox2 defined states
• Lineage-level dynamics are non-Markov over a time period of days to weeks
• A subset of lineages shows high transition rate between states
• These lineages skew cell fate under retinoic acid differentiation

## Graphical abstract

**Figure:**
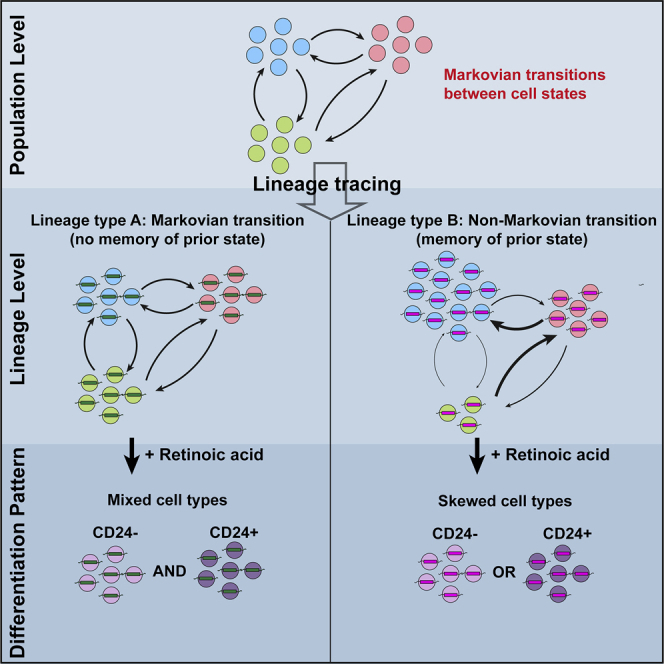

## Introduction

Stochastic processes have been described to play a role in multiple mammalian developmental pathways, ranging from hematopoiesis to fate choice of retinal progenitors (Boije et al., 2014; Till and Mc, 1961). For example, the development of mature retinal cell types from retinal precursor cells follows consistent probabilities as precursor cells choose a lineage fate without any apparent regard to environment or history and therefore has been termed stochastic (Losick and Desplan, 2008). In probability theory, a stochastic process that does not exhibit memory of its history is termed a Markovian process and is said to possess the Markov property. Therefore, for a memoryless (Markovian) stochastic process, the probability of visiting each state next depends only on the current state and not any preceding states. However, few studies of biological processes termed stochastic have formally assessed whether these processes possess the Markov property.

In biological development, cell states are often thought of as the expression of groups of genes at or near specific levels for each gene (Garg and Sharp, 2016). Knowing whether the history of a process influences future cell states is of particular interest for reversible transitions, where multiple paths could lead to the present state. Such reversible transitions occur in many contexts, such as maintenance of airway epithelium or intestinal crypts (de Sousa and de Sauvage, 2019; Nabhan et al., 2018; Pardo-Saganta et al., 2015; Tata et al., 2013; Tetteh et al., 2016), or in reprogramming experiments whereby differentiated cell types are induced to pluripotent cell states (Biddy et al., 2018). Understanding whether the history of prior states influences the probability of reaching particular future states will be important for understanding development and homeostasis of mammalian tissues.

One context in which to consider reversible state transitions is early embryogenesis in mammals, whereby loss of particular cells can lead to replacement through the developmental plasticity of neighbors (Chen et al., 2018; Martinez Arias et al., 2013). Embryonic stem cells (ESCs) provide an interesting model of early development, as these cells are derived from the inner mass of the blastocyst and can form all tissues of the adult vertebrate organism, and ESC state transitions in culture mimic developmental state transitions in embryos (Neagu et al., 2020; Shahbazi et al., 2017). ESCs show remarkable heterogeneity in the expression of key transcription factors, such as the pluripotency genes Nanog and Sox2 (Abranches et al., 2014; Chakraborty et al., 2020; Chambers et al., 2007; Filipczyk et al., 2015; Kalmar et al., 2009; Klein et al., 2015; Kumar et al., 2014; Singer et al., 2014; Ying et al., 2008), and heterogeneous expression in ESCs has been previously classified into discrete states with different developmental potential (Abranches et al., 2014; Filipczyk et al., 2015; Kalmar et al., 2009). ESCs dynamically interconvert between states, transitioning back and forth under standard culture conditions (Chakraborty et al., 2020; Filipczyk et al., 2015; Singer et al., 2014). Previous studies characterizing the dynamics of state transitions in this system have focused on states defined by levels of Nanog and have utilized fluorescent reporters in addition to antibody staining or fluorescence *in situ* hybridization (Chambers et al., 2007; Filipczyk et al., 2015; Singer et al., 2014). These studies have described the process of interconversion between states as stochastic, using measurements typically taken over timescales on the order of hours (Abranches et al., 2014; Hormoz et al., 2016; Ochiai et al., 2014; Singer et al., 2014). However, whether or not ESCs possesses the Markov property has not been extensively evaluated, and ESC state transitions over longer timescales have not been explored. Therefore, ESCs are a particularly interesting model system to consider memory of states, due to their ability to generate a diverse array of cell fates and their exhibiting reversible state transitions in culture.

One method to assess whether state transitions are a Markovian process is to examine the correlation between the cell states of daughter and cousin cells within a lineage of ESCs. In a Markovian process, each cell makes a state choice independent of its history, so the correlation of cell states between kin cells relaxes over time. A Markovian model thus predicts that eventually all cell lineages converge toward a consistent distribution of cell states as mixing amongst states occurs independently within each lineage. That is, for a stochastic memoryless process, all lineages should converge to the same distribution of states. Measuring how close or far a dynamical system is from this convergence point represents a type of informational entropy (Baez and Pollard, 2016). Whether or not ESC state transitions are Markovian processes and the degree to which they diverge from a Markovian model over long timescales is unknown.

Here, we characterize the dynamics of ESC state transitions amongst three interconverting states, defined by levels of Nanog and Sox2, which represent distinct gene expression programs related to development (Chakraborty et al., 2020). We genetically barcode ESCs, expand the population, and observe the proportion of each ESC lineage in each state over time. We find state history for ESC lineages influences future state transitions, and therefore, ESCs do not exhibit the Markovian property on the measured timescale for states defined by Nanog and Sox2 reporters. Surprisingly, a subset of ESC lineages shows concerted state transitions weeks after the barcode label is applied. These lineages show small but significant correlation in the amount of transition between replicate experiments. We measure the distribution of lineages across state space, compare them to the predictions of a Markov model, and quantify the difference as a type of informational entropy we term lineage entropy. Strikingly, the distribution of lineage entropy appears conserved over time. Finally, we show that lineages with a high frequency of concerted state transitions are more likely to skew their cell fates into neuroectoderm or extraembryonic endoderm when cultured under differentiation conditions. Together, these data show ESC transitions between states, defined by levels of pluripotency gene reporters, do not possess the Markov property and highlight the role of ESC lineage in determining cell state path and differentiation outcomes.

## Results

### Generation and tracking of ESC lineages over time

**Figure 1:**
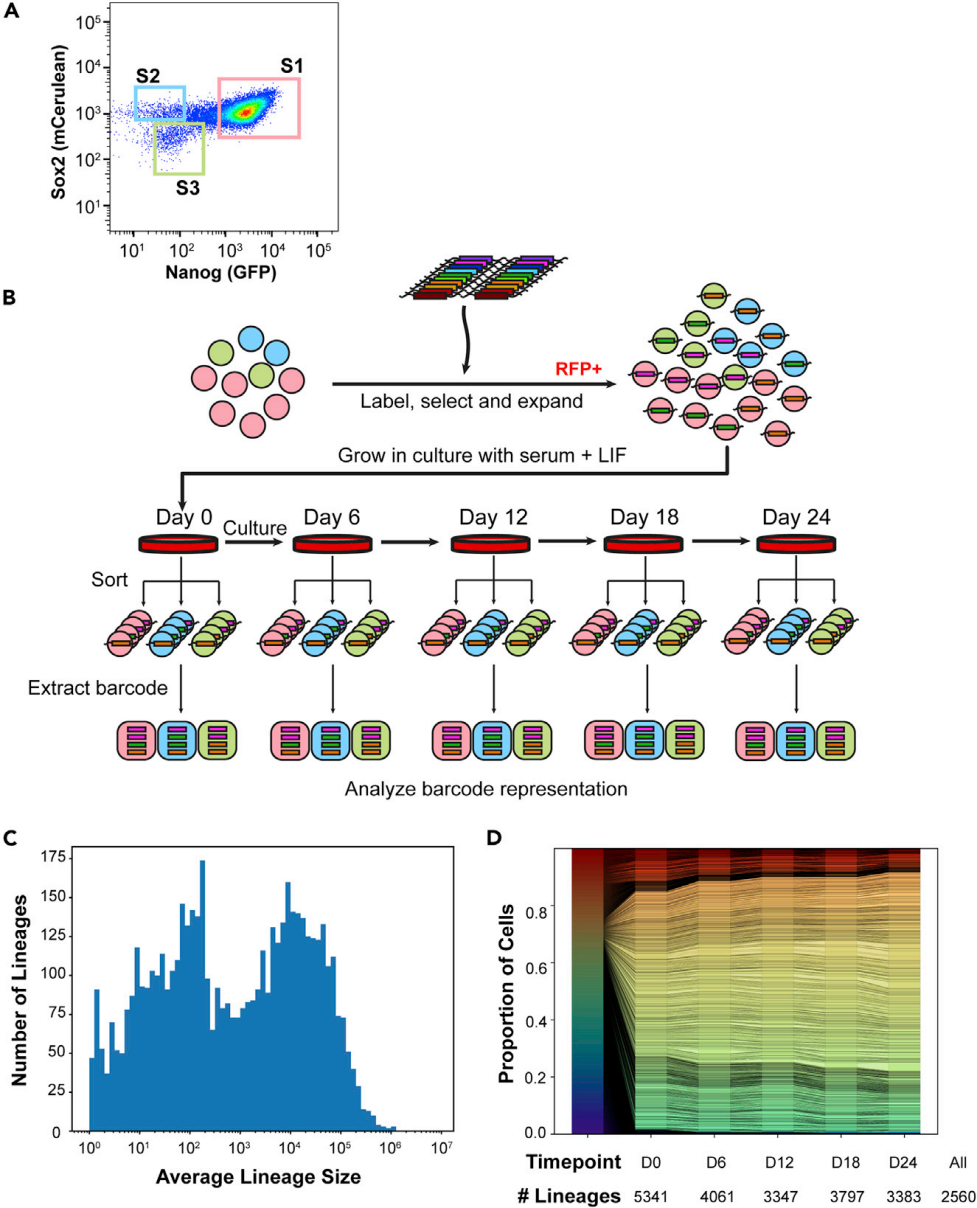
Generation and tracking of ESC lineages (A) FACS plot showing the expression of Nanog and Sox2 in a population of mouse embryonic stem cells. Cells were binned into three states of expression (State 1 = Nanog High Sox2 High, State 2 = Nanog Low Sox2 High, State 3 = Nanog Low Sox2 Low). (B) Experimental Schematic. Lentivirally encoded barcodes were introduced into ESCs in the three States which were expanded into lineages. Cells were cultured over time, during which some ESCs switched between States. At the indicated time points half the culture was sorted into States 1–3 and the representation of each barcode (lineage) assessed in each state through sequencing (see STAR Methods). Pink circles, Blue circles and Green circles represent State 1, State 2 and State 3, respectively. See Table S1 for cell numbers assessed. (C) Histogram showing the distribution of lineage sizes (in number of cells) on average across all time points. (D) Stacked bar plot representation showing the proportion each lineage contributed to the overall population across all time points. Each unique color row represents a distinct lineage. The number of lineages observed above background at each time point is indicated; 2,560 lineages were detected in at least one state at all time points. See also Figures S1 and S2.

To assess the dynamics of ESC state transitions over time, we constructed an ESC reporter line compatible with barcoding and state readout. We generated ESC with heterozygous insertions of fluorophore tags at the endogenous loci of *Nanog* and *Sox2* (*GFP*-P2A-*Nanog* and *Sox2*-P2A-*mCerulean3* respectively, Figure S1A). We previously divided these cells into three predominant states of Nanog and Sox2 expression (State 1 = High Nanog and High Sox2, State 2 = Low Nanog and High Sox2, and State 3 = Low Nanog and Low Sox2; Figure 1A and [Chakraborty et al., 2020]) in ESC. We transduced ESCs with a lentiviral barcoding vector (Bhang et al., 2015) at a low multiplicity of infection, ensuring each cell received ≤ 1 barcode (Figure S1B). After selecting for ∼100,000 transduced, labeled cells representing at least 5,341 distinct barcoding events, we expanded the population for nine days to 10^8^ total cells. This allowed each barcoded ESC the chance to expand to an estimated ∼15,000 cells (95^th^ percentile range: 17–103,877 cells, interquartile range of 16,092) distributed across all three ESC states, which were continuously cultured together (Figure 1B). We refer to these expanded, single ESC-derived cells as ESC lineages since the incorporated lentiviral barcode will be copied in each progeny cell, marking all ESC with the same unique barcode as kin.

We cultured these ESC lineages together and assessed their distribution across the three ESC states over a period of 24 days (Figure 1B). First, we split our culture of 10^8^ cells, maintaining half in culture and isolating State 1, State 2, and State 3 cells from the other half by flow cytometric sorting for the fluorophore markers of Nanog and Sox2. Gates used for sorting populations of States 1–3 are shown (Figure S2A) and were chosen to minimize cross contamination between state populations. After day 0, the cultured ESC population was maintained at ≥ 2×10^7^ cells at all times to ensure high representation of lineages and was split either every day or every other day due to the rapidly dividing nature of ESCs under standard culture conditions (doubling time ∼12–14 h). Consistent with a dynamic equilibrium between states and our previous experience (Chakraborty et al., 2020), the proportions of ESC within States 1–3 remained relatively constant over time (Figure S2B). We assessed the distribution of ESC lineages across the States in a similar manner by sorting at least 5×10^7^ cells every 6 days (Figure 1B), isolating ∼2–4×10^5^ cells for each state at each time point to ensure representation (see Table S1 for cell numbers). We then identified the number of cells for each lineage in each state through the relative proportion of each barcode in each sample (Figure S2C and STAR Methods). We confirmed lineages were adequately detected through subsampling the data and noting a minimal effect on the size distribution of lineages detected (Figure S2D). The estimated number of cells in each ESC lineage is shown (Figures 1C and S2C and STAR Methods). A total of 2,560 lineages were confidently identified in at least one state at all time points of the experiment and are the focus of subsequent lineage level analysis.

Interestingly, the distribution of lineage sizes did not change appreciably over time (Figure S1) nor did any particular lineages come to dominate the mixed culture by size (Figure 1D). This is in contrast to lineage competition in other biological systems, such as cellular reprogramming or differentiation, in which particular clones dominate the population (Chan et al., 2019; Shakiba et al., 2019). Our system reliably allowed us to track thousands of ESC lineages and their distribution across states over an extended period of time.

### Fitting of a Markov transition matrix to ESC State changes

**Figure 2:**
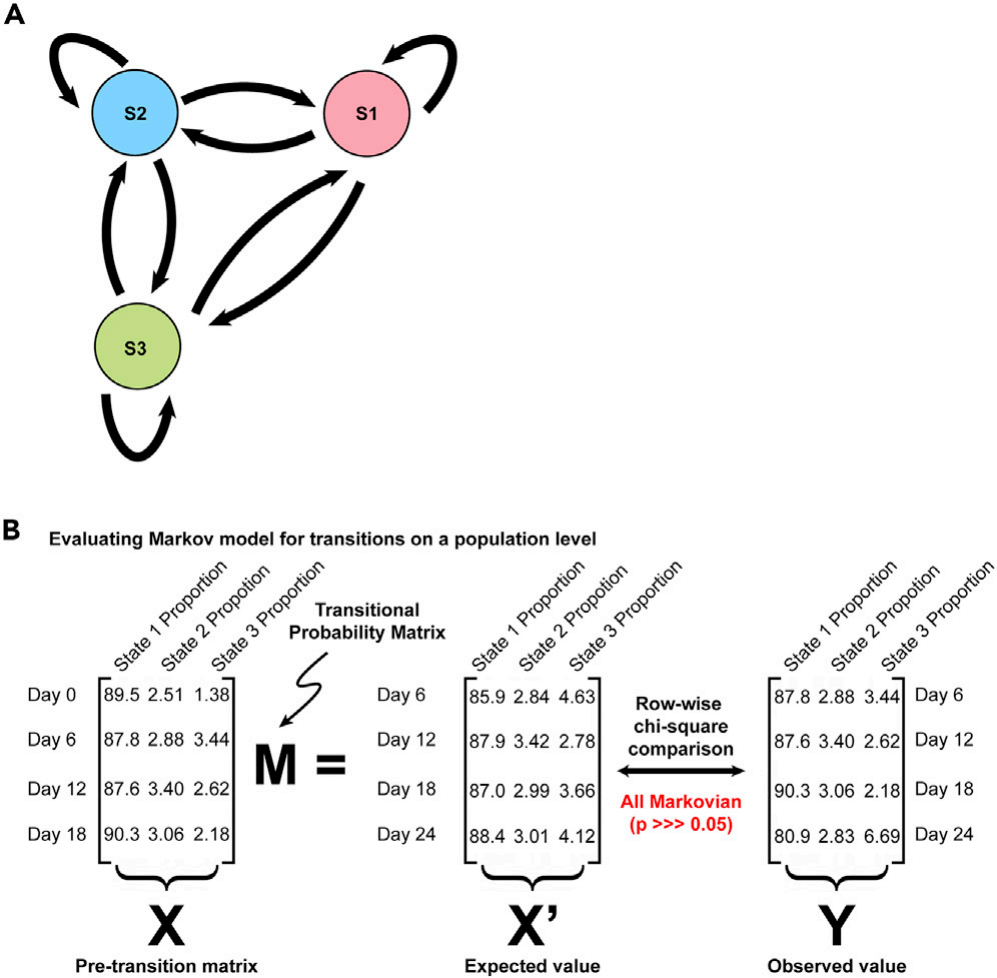
ESC population dynamics fit a 3-state Markov model (A) Schematic of a Markov model with transitions between all 3 states. (B) Framework for considering whether proportions of ESC in States 1–3 fit a 3-state model. The proportion of ESC in each state prior to transition was compared to the proportion of ESC in each state after transition to fit a transition probability matrix. This matrix predicted state proportions after each transition that were not different from the observed proportions (row-wise chi-square test, p > 0.05), indicating the population of ESC as a whole was at equilibrium and could be well fit by a Markov model. See Figure S2B for flow cytometry data giving the proportion of population in each state.

We noted that the overall population of ESC maintained a relatively consistent composition between States 1–3 over time (Figure S2B). Thus, first we determined whether the overall population of ESC transitions was well fit by a 3-state Markov model (Figure 2A). We used the proportion of the population in each state before transition (X, at days 0, 6, 12, and 18) compared to the proportion of the population in each state after transition (Y, at days 6, 12, 18, and 24) to solve for a transitional probability matrix M**,** using linear least-square estimation (Figure 2B, Notes):

(Equation 1) XM=Y

If this transitional probability matrix, calculated using transitions across all five time points, could predict each individual transition's proportions accurately, then the system overall would be considered well fit by the Markov model. Thus, next we applied M to the pretransition population frequencies in States 1–3 at each time point (pretransition matrix X, Figure 2B), which yielded the expected frequencies for States 1–3 after transition predicted by the Markov model (expected values $X^{\prime }$, Figure 2B). We compared these to the actual observed frequencies in each state after transition (observed values Y, Figure 2B) and found no significant differences between the expected values predicted by the Markov model and the observed state proportions as evaluated by chi-square ($\chi^{2}$) testing. Consistent with previous studies (Abranches et al., 2014; Filipczyk et al., 2015; Ochiai et al., 2014; Singer et al., 2014), we conclude that ESC state transitions are well fit by a 3-state Markov model when the entire ESC population is considered together.

### Distribution and transitions of ESC lineages over time

**Figure 3:**
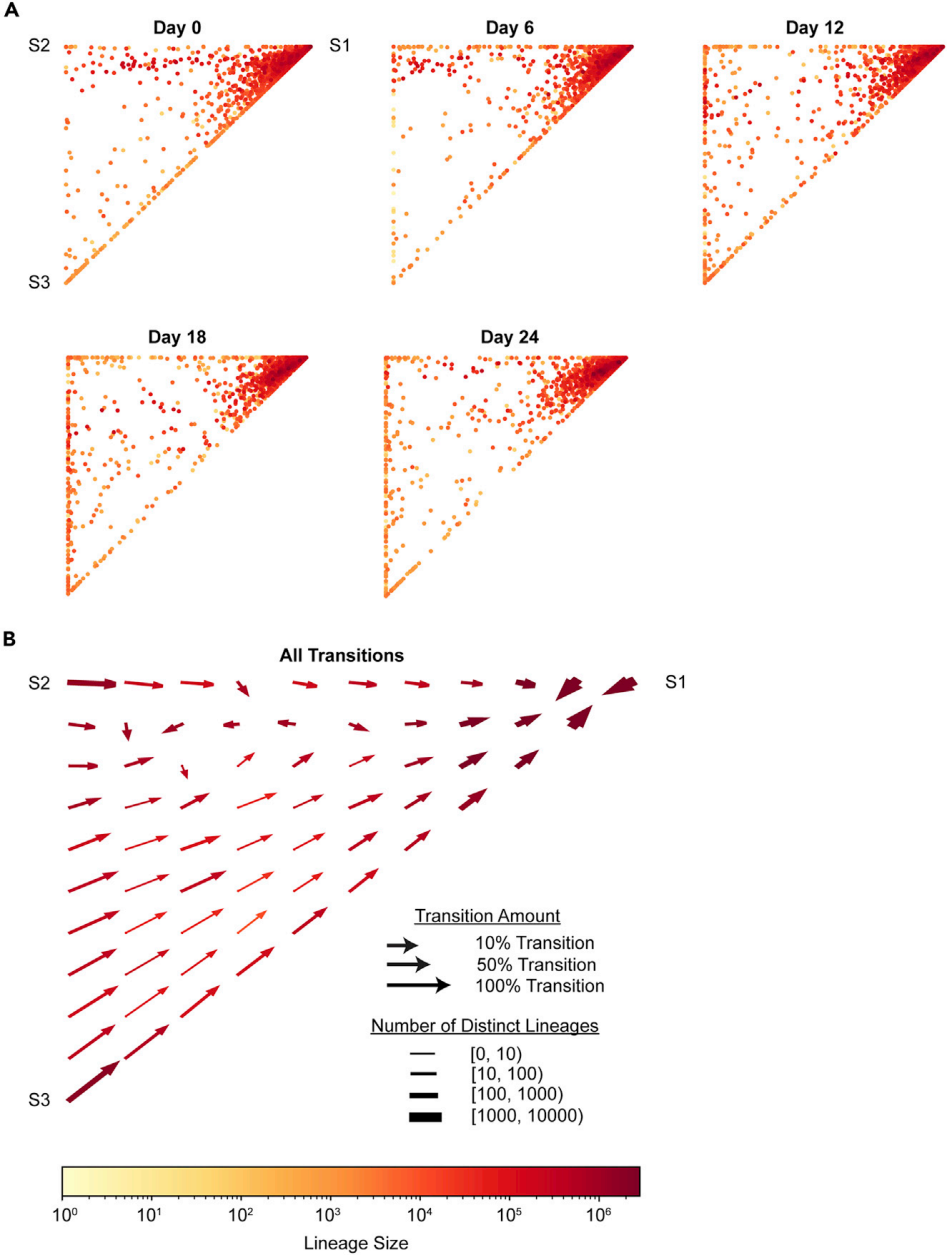
Distribution of ESC lineages over time (A) Ternary plots showing the proportion of each lineage across states for all ESC lineages over time. Lineages in the corners were detected as present only within that one state. (B) Vector field showing the local average change in state proportions between two contiguous time points (such as day 0 → day 6, representing a possible state transition). The local averages for all four transitions (day 0 → 6, day 6 → 12, day 12 → 18, day 18 → 24) were equally weighted. See also Figure S3.

While a 3-state Markov model fits ESC population dynamics, this observation can conceal different underlying dynamics for individual ESC lineages. In order to test the Markov model on individual ESC lineages, first, we assessed the dynamics and distribution of cell state for each ESC lineage. We calculated the fraction of cells in each state and represented these data on a ternary plot in which each dot is a lineage and its position indicates the relative composition of State 1, State 2, and State 3 in that lineage at each time point (Figures 3A, S3A, and S3B). For example, a lineage on the top right corner of this plot indicates that all cells were in State 1 and the lineage was not detected in the States 2 or 3 samples, and analogously a lineage on the bottom left or top left corner indicates a lineage only present in State 3 or State 2, respectively. As expected, the majority of cells in most lineages were in State 1 (Figure 3A). Additionally, many lineages (955 of 2,560) were detected in all three states across all time points (Figure S3A). At all time points, a subset of lineages was detected as present only in one or two states, as evidenced by the continued presence of lineages at or near the edges of the ternary plot.

Next, we sought to understand the dynamics of how cells in each lineage transitioned between states over time. First, we considered the change in proportion of each state for each lineage as a vector between two points on the ternary plot, and generated a vector field diagram. The diagram represents the summated transitions of all lineages present in each location of the plot: the fraction of lineages transitioning, the total size of cells, and the number of distinct lineages making transitions are all displayed (Figure 2B). This plot was fairly constant for all four transitions captured in our experiment (day 0 → 6, day 6 → 12, day 12 → 18, and day 18 → 24, Figure S3C). The vector field plot revealed the overwhelming tendency of lineages present in the State 2 or 3 corner of the ternary plot to return to State 1 at the next time point and for lineages located in the State 1 region of the plot to switch into States 2 and 3. This is in agreement with previous studies showing individual ESC transitioning between Nanog-high and Nanog-low states (Filipczyk et al., 2015; Singer et al., 2014), though these studies traced ESC on a shorter timescale of hours compared to the present study. However, the observation that ESC lineages also show net transitions between State 1 and States 2 and 3 at later time points is surprising, as cells transitioning in and out of a particular state might be expected to cancel out, leaving the lineage as a whole with no net change in position.

**Figure 4:**
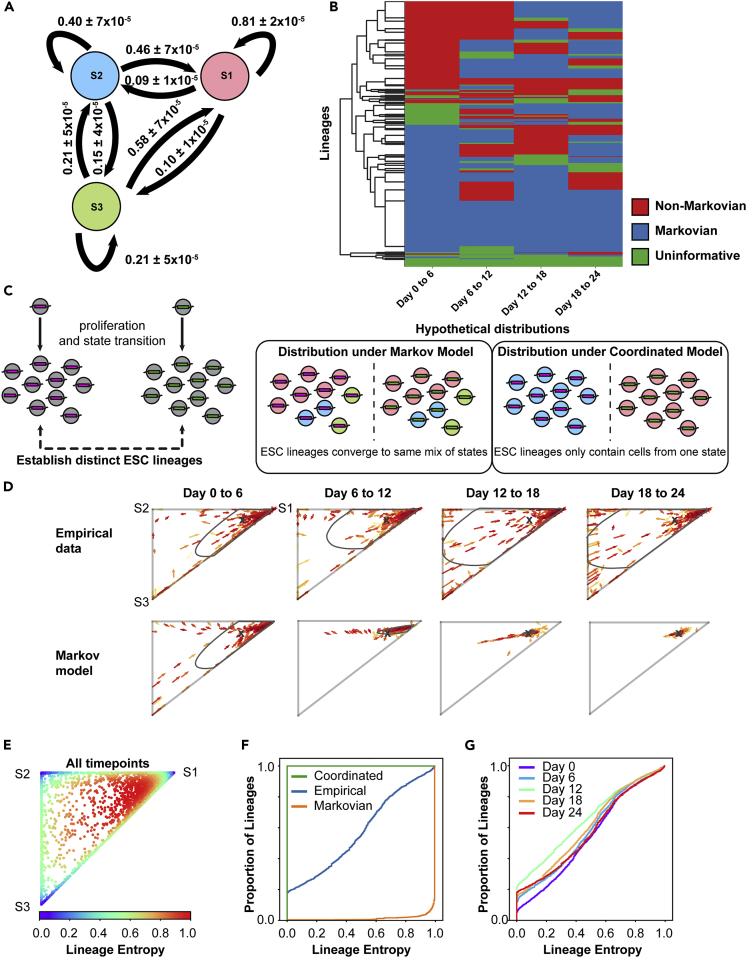
Some ESC lineages exhibit coordinated, non-Markov transitions between states (A) Overall transitional probabilities between states inferred in the ESC system (see Note). Pink circle, blue circle, and green circle represents State 1, State 2, and State 3, respectively. Confidence intervals represent 95^th^ percentiles of bootstrapping. (B) Lineages (rows) were evaluated as fitting Markovian or non-Markovian dynamics using lineage specific transition probability matrices and chi-squared test of homogeneity. Due to occupancy of only one state, some transitions are uninformative. Transitions for 1,736 of 2,560 lineages are displayed; the remainder had low cell number for this analysis (Note). Hierarchical clustering of transition patterns is shown. (C) Schematic of state transitions under two contrasting models. In the first, ESCs are assumed to possess the Markov property and are agnostic to their history, therefore over time, ESC lineages converge to the same mix amongst states and same point on the ternary plot. In the second, ESC transitions are determined by their history and kin relations, therefore ESC lineages exhibit coordinated transitions and converge to the corners of the ternary plot. (D) Arrow plots show the transition of a subset of lineages from both the empirical data and that predicted by the Markov model. Arrow color indicates lineage size (scale matches that used in Figure 3). Gray boundary indicates 95^th^ percentile of lineage location (empirical data) or 95% confidence interval for prediction of Markov model. X marks the equilibrium point predicted by Markov model. (E) Ternary plot displaying the lineage entropy (informational entropy) value for all lineages from all time points. (F) Cumulative distribution function (CDF) plot showing lineage entropy distribution for coordinated and Markovian hypothetical models (green and yellow, respectively) and the empirical data from D (blue). (G) CDF plot showing the distribution across all ESC lineages of lineage entropy for the empirical data at different time points.

The information of how each individual ESC lineage transitioned between time points allowed us to calculate additional matrices encompassing transitional probabilities in this system. For each transition, we considered the proportion of each lineage in each state at time t_n-1_ as $\vec{X}$ and those in each state at time t_n_ as $\vec{Y}$. This allowed us to solve for the transition matrix M given by:

(Equation 2) $$\vec{X}M=\vec{Y}$$

using least-squares estimation (see Note). This matrix M represents a Markov transition matrix fit based on average observed dynamics over data derived from the entire time course experiment across all lineages. Transitional probabilities between the three states are shown (Figure 4A), and confidence intervals for the parameters of M were estimated by bootstrapping (Figure S4A, Note). Next, we used this transition matrix (M) along with the known real sizes of each lineage (Figure S1) before and after transition to calculate the net change in cell number for each type of transition (e.g., State 2 → State 1, State 1 → State 3, State 2 → State 2, etc.) on average across all lineages. This rate of change represents a net combination of growth, birth, and death events for cells making each type of transition or staying within their state (Figure S4B, see also matrix G in Note). Interestingly, cells transitioning from State 1 to State 3 show a net growth-birth-date rate of 21%, meaning cells making this transition show much smaller apparent population sizes after transition. In contrast, cells making the reciprocal State 3 to State 1 transition show a net growth-birth-death rate of 847%, indicating they greatly increased in cell number. Together, the rates of state transition and growth-birth-death between States 1 and 3 constituted a description of the dynamics in this system on average across ESC lineages.

### Path dependence and violation of a memoryless (Markovian) assumption

Next, we sought to analyze whether individual ESC lineage transitions had the Markov property. We fit each of the 2,560 lineages by a transition matrix by comparing its pretransition distribution amongst States 1–3 to its post-transition distribution amongst States 1–3, similar to the process performed for the entire ESC population (Figure 2). The state distribution (conditional probabilities) for each lineage across time points is given (Table S2). For each lineage, we compared its expected distribution across each transition (e.g. day 0 → day 6) to its actual distribution after transition using chi-square ($\chi^{2}$) testing. We classified transitions as Markovian if the lineage-specific transition matrix ($M_{i}$) fit the transition and non-Markovian if there was a significant deviation between the expected and observed values. For these comparisons, a number of transitions were uninformative because cells in the lineage were present in only one state at a given transition or the lineage was too small in size for statistics (see Note). Overall, 1,183 lineages showed at least one non-Markovian transition and 114 lineages showed all non-Markovian transitions (out of 1,736 total) across the experiment (Figure 4B). Distributions for the Markov transition matrix parameters ($M_{i}$) for the 114 lineages most out of equilibrium are shown (Figure S4C). Thus, analysis of ESC lineages contrasted with the analysis of the ESC population as a whole irrespective of lineage, where the population was well fit by a Markov transition matrix (Figure 2), and suggested dynamics might differ within individual ESC lineages.

While transitions between states happen at the level of cells, if there is memory within a lineage there could also be concerted transitions at the level of the whole lineage. Thus, in addition to evaluating whether the distributions of cells amongst states fit a Markov model, we analyzed the sequence of states occupied by each lineage over time. We consider a lineage in a given state (States 1, 2, or 3) if a plurality of cells in that lineage occupy the state; in other words, for each lineage whichever state contains the highest proportion of cells is defined as the state of that lineage. A stochastic process is said to possess the Markov property if for the set of variables under consideration X (in our case, the group of ESC lineages) occupying states given by S (States 1, 2, and 3):

(Equation 3) $$\text{Probability}\left(X_{n+1}=s|X_{1}=s_{1},X_{2}=s_{2},\ldots ,X_{n}=s_{n})=\text{Probability}(X_{n+1}=s|X_{n}={\text{s}}_{n}\right)$$

where $s_{n}$ are the states of each lineage at time point n (days 0, 6, 12, 18, and 24). Stated, this means that the distribution of X (lineages across states) at the next time point depends only on the present state and not the entire history of transitions (see Note). In other words, the Markov property means where a lineage transitions next depends only on where it is now and not where it has been previously. We enumerated the probability of transitions between states for all 2,560 ESC lineages (Table S2) to assess this statement. Strikingly, several lineages showed highly divergent conditional probabilities when the entirety of their history was considered (Table S2). For example, we compared two lineage histories that were both in State 2 on day 18 and assessed their probability of remaining in State 2 on day 24. In the first history, lineages that were in State 1 on days 0, 6, and 12 showed only a 21 percent probability of remaining in State 2 on days 18 → 24. However, this probability rose to 64 percent in the second history, where lineages were in State 2 on days 0, 6, and 12 (p-value 1.47×10^−6^, Fisher's exact test). We confirmed these patterns were not dependent on the plurality vote threshold used to determine the state of a lineage, as changing the criteria for state membership did not largely impact the number of non-Markovian sequence motifs (Figure S5). In a related analysis, we generated recenter plots to visualize lineage sequence over the course of the experiment. These plots showed patterns whereby cells followed together through several transitions along a specific path between States 1 and 3 (Figure S6 and Table S2). The course of lineages through the experiment was also visualized using a probability decision tree matrix (Figure S7). Altogether, transitioning lineages were heavily biased to transition between States 1 and 2 or between States 1 and 3, with relatively few mixing transitions between States 2 and 3. This is consistent with the idea that States 2 and 3 represent distinct gene expression programs related to developmental time points downstream of State 1 (Chakraborty et al., 2020).

Further, we inferred transitions at the level of individual cells. If ESC state transitions possess the Markov property at the level of individual cells, distinct lineages of related ESCs should converge to the same distribution across states as every cell makes a separate choice of state regardless of its history and, therefore, its kin relations. This is equivalent to the idea that in a Markov process, each cell will sample from the same underlying probability distribution when choosing its next state. Thus, we compared ESC lineages over time under a Markov model with a coordinated model in which related cells remain more likely to occupy similar states at later time points (Figure 4C). We visualized the dynamics of lineages transitioning under both models (Video S1 and Video S2). A Markov model did not capture the dynamics of lineages in transitioning between states, as at all time points, some lineages were distributed away from the equilibrium point and others appeared to be transitioning away (Figure 4D, equilibrium point and 95^th^ percentiles for empirical data and 95% confidence intervals for predictions of the Markov model are marked, see also Figures 3A and S3C). Additionally, a fully coordinated model did not capture the data as not all lineages were distributed on the corners of the plot, with most lineages containing cells in each state. Instead, the system appeared to contain a mix of ESC lineages retaining information about their kinship history and transitioning together and other lineages that were either not transitioning between states or had relatively equal numbers of cells making reciprocal transitions (i.e., one cell of the lineage transitions State 2 → 1, while another cell transitions State 1 → 2 such that the net proportion of the lineage in each state remains unchanged). Our system did not allow us to distinguish between these two possibilities. Nevertheless, together with assessment of state transition probabilities this analysis demonstrated ESCs do not transition between Nanog- and Sox2-defined cell states in a completely memoryless manner. Rather, at least a subset of ESCs retains information about past states that influences future transitions.

Video S1. Animation shows how lineages empirically change their state distribution over 5 timepoints, related to Figure 4C Each dot represents a lineage colored by its overall motility across 4 transitions

Video S2. Animation shows how lineages change their state distribution over 5 timepoints under a Markov model, related to Figure 4C Each dot represents a lineage colored by its overall motility across 4 transitions.

### Informational entropy in ESC lineages

Measuring how different lineages of cells distribute across state space represents a measure of information contained in the system. We sought to quantify the information retained by the system of ESC lineages. Compared to equilibrium where all lineages become perfectly mixed in their proportion of states over time, the informational gain can be thought of as the relative information, information entropy, or relative entropy; we will use the term lineage entropy in the present context. All of these terms represent a quantity that approximates how far the system is from maximal uncertainty, which is achieved when all lineages are at a perfectly mixed equilibrium point. In the scenario of a Markov process, convergence of all related cells in an ESC lineage to an equilibrium distribution across states represents maximal lineage entropy. Conversely, ESC lineages where all cells are in the same state would represent minimal lineage entropy. We quantified the relative entropy of each lineage compared to the equilibrium point using a modified version of the Shannon entropy ([Baez and Pollard, 2016], see Note). The lineage entropy at each point in the ternary plot is shown (Figure 4E).

Next, we compared lineage entropy in our empirical data with that of the Markov model and a coordinated model, representing the data by plotting the cumulative distribution of lineage entropy across all lineages (Figure 4F). We found the empirical distribution of lineage entropy diverged significantly from either model. More interestingly, the distribution of lineage entropy appeared relatively unchanged over the time course of the experiment (Figure 4G). Maintaining the distribution of lineage entropy throughout the experiment is unexpected as the informational entropy in a stochastic system labeled at one distinct time point (introduction of barcodes) would be expected to strictly increase as the labels become diluted over time. Together with the analysis of transition probabilities, this suggests lineage history is a necessary variable to take into account when predicting the future state of the ESC system, independent of the present state.

### Defining ESC state by transition probability instead of gene expression

**Figure 5:**
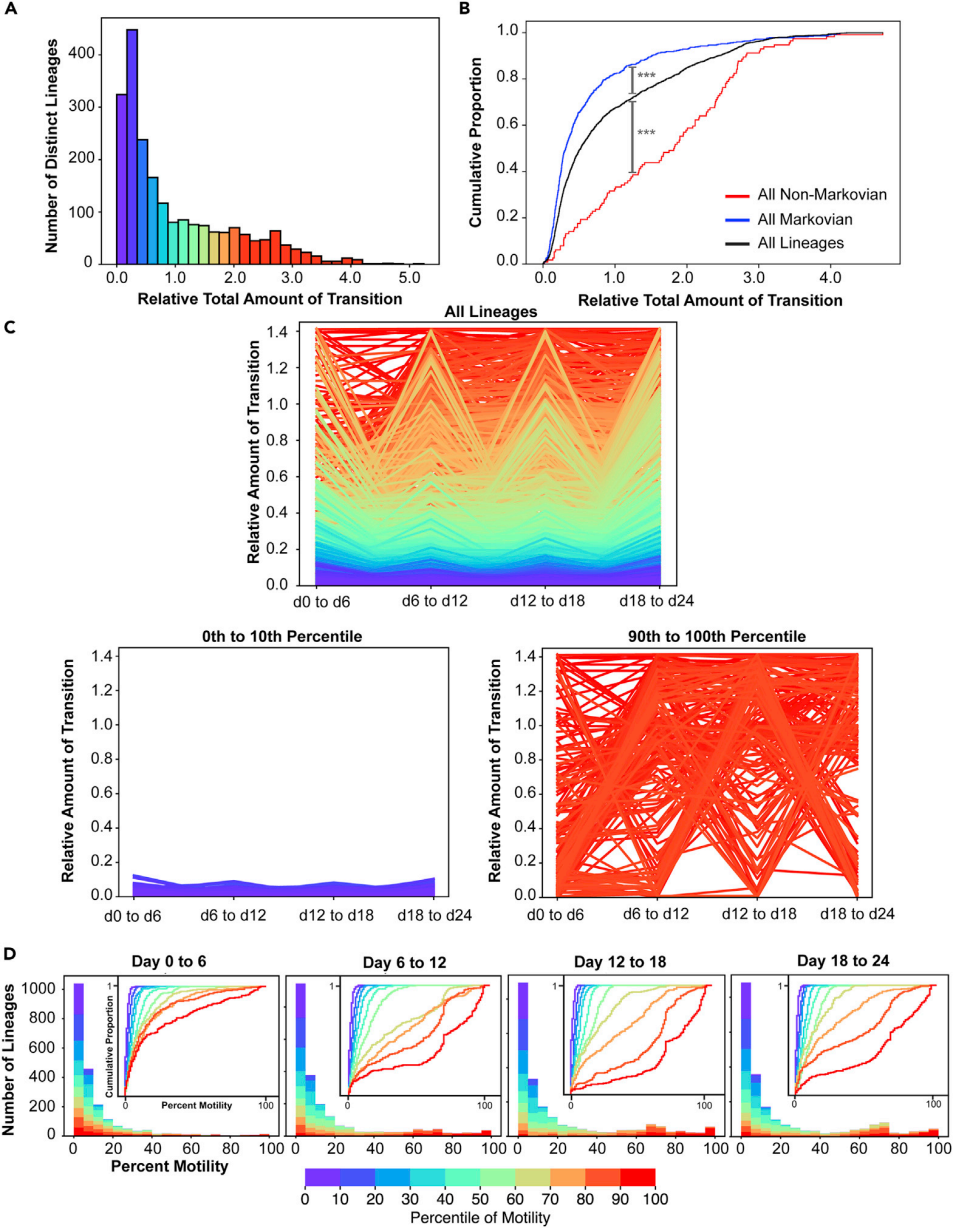
A consistent subset of ESC lineages is characterized by a high amount of state transitions (A) Histogram showing the number of lineages with differing amounts of transition between states. Lineages are colored according to their percentile rank of their motility between states across all time points relative to all 2,560 measured. (B) Cumulative distribution function (CDF) plot showing the relationship between memory and motility. The relative total amount of transition is shown for all lineages, and compared to lineages with all Markovian or all non-Markovian transitions. p < 0.001 for comparisons to all lineages, Kolmogorov-Smirnov test. (C) Line chart showing the amount of transition for all lineages at each transition point. Line charts showing the amount of transition for lineages in the first decile (left) and the last decile (right) of motility are highlighted below. (D) Stacked bar plots show the distribution of percent motility from lineages in different decile groups of transition. Inset: CDF plot providing additional visualization of distribution of percent motility. In A–D, all lineages are decile ranked according to their cumulative overall motility across transitions and colored identically in each panel.

In examining the transitions of ESC lineages between states, we noted that most lineages fell into one of two categories: either they exhibited concerted transitions between State 1 and States 2 and 3 (Video S1, red lineages) or they exhibited no net transitions at all (Video S1, blue lineages). This led us to consider whether lineages might be properly classified on the basis of whether they were highly dynamic and exhibited concerted transitions between states (high “motility”) rather than their levels of gene expression. We calculated the motility of each lineage as the total distance traveled on the ternary plot over time (see STAR Methods). First, we addressed whether the same lineages were transitioning into and out of State 1 over time. A subset of highly dynamic lineages displayed high motility in transitioning between states (Figures 5A and S8A). These lineages showed a range of sizes, containing a few to thousands of cells, but did not include the largest sized lineages, which may have been at equilibrium (Figure S8B). Next, we compared motility on the ternary plot to the Markov or non-Markov nature of each lineage when evaluated by its lineage specific transition matrix ($M_{\text{i}}$, Figure 4B, above). We plotted a cumulative distribution of motility across all lineages, lineages with only Markovian transitions (all blue in Figure 4B) or all non-Markovian transitions (all red in Figure 4B). We found non-Markovian lineages were skewed toward higher motility and Markovian lineages toward lower motility, consistent with the former being further away from the equilibrium point (Figure 5B, p < 0.001 Kolmogorov-Smirnov test).

Analyzing motility of all lineages over time demonstrated a consistent subset transitioning between states, resulting in a “sawtooth” type appearance when motility was plotted against time (Figures 5C and S9A). We confirmed highly dynamic lineages were not due to sampling a portion of cells in states by considering the change in motility as data were subsampled (Figure S9B), which was minimal. To visualize this effect in another fashion, we plotted the portion of each lineage transitioning at each time point and colored lineages by their overall transition amount across the whole experiment (Figures 5D and S9C). The consistent “skew” of red, highly dynamic lineages to the right indicates that this same group of lineages was transitioning between states at each time point. Finally, we calculated the pairwise correlation of motility for each lineage across transitions, which confirmed correlation between motility at early transitions and later transitions (Pearson's *r* ranging from 0.19 to 0.50 Figure S10A). We assessed whether these effects could be due to random amplification during library preparation by analyzing base frequency in high motility lineages, which was not skewed, and by randomly reassigning read counts, which resulted in no motility correlation between early and late transitions (Figure S11).

This led us to consider whether motility was a conserved feature defining state in the ESC system, and if states would be better considered as “motile” lineages and “nonmotile” lineages irrespective of specific Nanog and Sox2 levels. When dividing lineages into motile and nonmotile states, we again did not observe the Markovian property, as high motility at all prior transitions was associated with a higher probability of remaining high in motility when compared against a lineage that was only high in motility at the immediately preceding transition (Table S3 and Figure S12 and Note). To further elucidate whether motility of a lineage was a conserved feature, we performed a repeat of our entire experiment in duplicate (Figure S10B) and compared the motility of each lineage at each transition between replicate experiments, comparing lineages with the same barcode in each replicate to each other. We found a small correlation (Pearson's *r* ranging from 0.15 to 0.31) between motility across replicates (Figure S10C), which was significant when compared to a model in which each lineage sampled its motility randomly from the experimental distribution (Figure S10D) but less than the average correlation of motility within each replicate (Pearson's *r* ranging from 0.19 to 0.50). Additionally, both replicates demonstrated non-Markovian state transitions and conservation of lineage entropy (data not shown). Together, we conclude that motility between cell states shows modest correlation across transitions and replicates in the ESC system.

### Non-Markovian lineages skew fates upon ESC differentiation

States 1–3 represent interconverting, metastable gene expression states in ESC under culture conditions. The identification of a subset of ESC lineages with high rate of concerted, non-Markovian transitions between States 1–3 raised the question of whether this property impacted ESC differentiation. To address this question, we utilized retinoic acid treatment of ESC, which leads ESC to stop dividing and differentiate into a population representing neuroectoderm and extraembryonic endoderm cell types (Niakan et al., 2013; Semrau et al., 2017; Ying et al., 2003). After retinoic acid treatment, these cell types can be separated by flow cytometric sorting using levels of the cell surface marker CD24 (CD24^high^ = neuroectoderm, CD24^low^ = extraembryonic endoderm). We confirmed retinoic acid treatment of ESC led to upregulation of neuroectodermal markers in the CD24^high^ population and upregulation of extraembryonic endoderm markers in the CD24^low^ population (Figures S13A and S13B).

**Figure 6:**
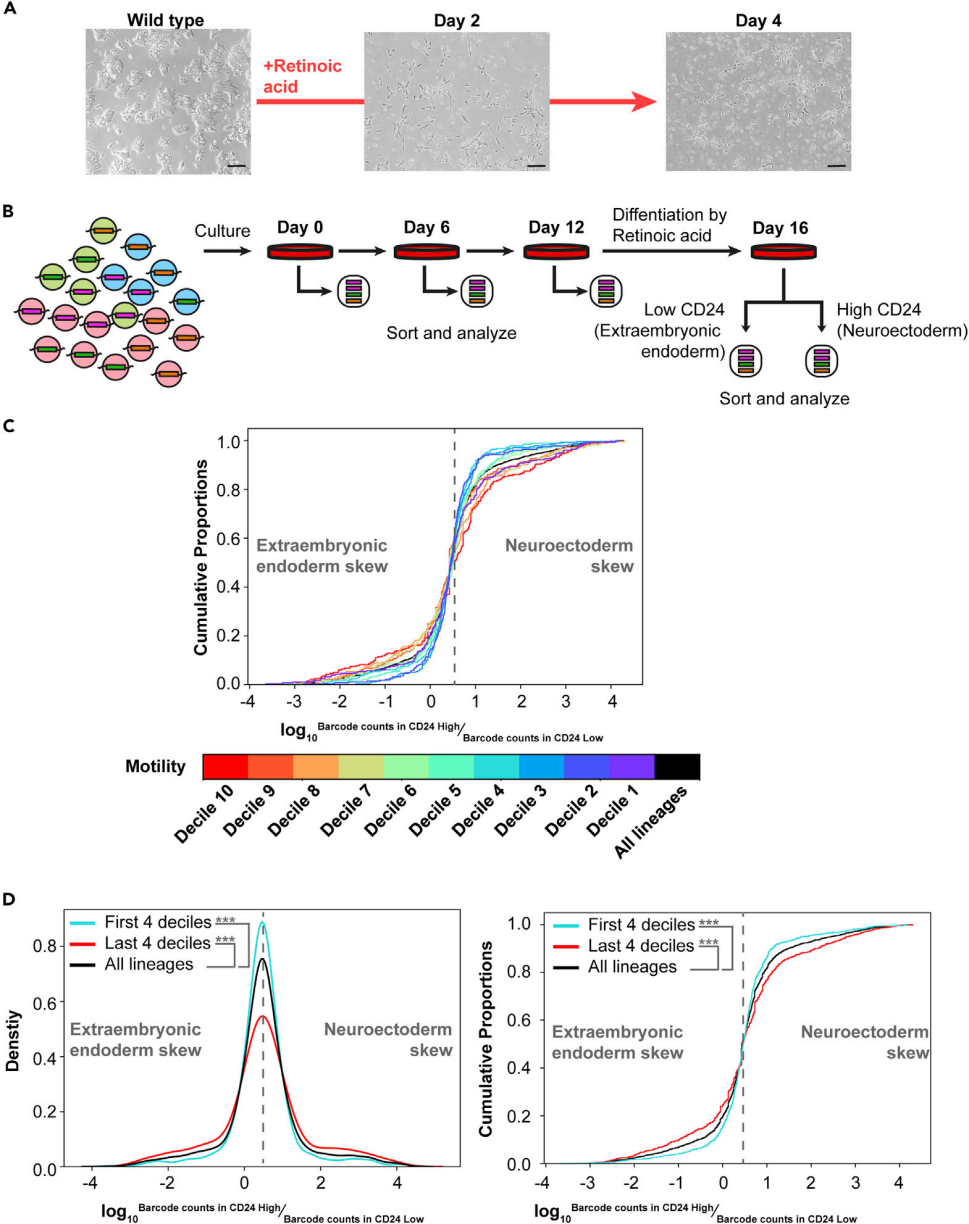
High motility ESC lineages skew fate to either neuroectoderm or extraembryonic endoderm upon differentiation (A) Phase contrast microscopy of embryonic stem cell colonies in standard culture, differentiated by 4 days of treatment with retinoic acid. 10X objective images. Scale bar = 25 micrometers. (B) Diagram of experiment. ESC lineages were cultured under standard conditions (serum + LIF) under which cells transition between States 1–3 for 12 days, with sampling on day 0, 6, and 12. On day 12, a cell split was also placed in retinoic acid for differentiation. CD24^high^ (neuroectoderm) and CD24^low^ (extraembryonic endoderm) cells were isolated by flow cytometric sorting and the lineages (barcodes) in each population assessed by sequencing. (C) For each lineage, the ratio of its occurrence in CD24^high^ to CD24^low^ cells is plotted (CDF). Lineages are separated into deciles of motility across days 0–12. F-test for variance p < 0.01 for all deciles compared against all lineages except for the fifth decile, which was not significant. (D) As in part C, except the top 4 deciles of motility and bottom 4 deciles of motility are grouped to enable visualization. A histogram (left) and CDF plot (right) are shown. F-test for variance p < 0.001 for both groups compared to all lineages.

Next, similar to the experiment performed above (Figures 1, 2, 3, 4, and 5), we cultured the ESC lineages under standard conditions with interconversion between States 1–3 for 12 days, allowing us to identify high motility lineages (Figure 6B). Then, we differentiated ESC lineages for 4 days in retinoic acid, separated CD24^high^ (neuroectoderm) from CD24^low^ (extraembryonic endoderm), and assessed the lineage (barcode) representation in each population. For each lineage, we calculated its ratio between CD24^high^ and CD24^low^ populations and plotted a cumulative distribution over this ratio. We found higher motility lineages were skewed in their proportions, either showing relatively greater numbers of neuroectoderm (CD24^high^) or extraembryonic endoderm (CD24^low^) cells (Figure 6C, p < 0.01 for all deciles except the fifth, F-test for variance). Lineages that were detected in States 2 or 3 by plurality vote were also skewed, consistent with the idea that these states may be primed for differentiation and consistent with differences in their gene expression profiles (Figure S13B and Chakraborty et al., 2020). Conversely, low-motility lineages were more likely to be evenly split between neuroectoderm and extraembryonic endoderm fates, perhaps reflecting the fact that these lineages were more likely to be in equilibrium between States 1 and 3 prior to the addition of differentiation signals. We visualized these differences by combining the top and bottom 4 deciles of motility (Figure 6D, p < 0.001 for both groups, F-test for variance). Together, these data indicated that a subset of non-Markovian ESC lineages was poised to transition between states and skew their fate upon addition of a strong differentiation signal.

## Discussion

We analyze lineages of ESCs transitioning between Nanog- and Sox2-defined states over days and find a subset of lineages with cells that transition between states together. These lineages appear to follow distinct, specific paths of state transitions, with the full history of the lineage influencing the probability of future transitions. Therefore, we deduce at least some ESC lineages do not possess the Markov property of memorylessness. When exposed to a strong differentiation signal, non-Markov lineages show greater skew in cell fate.

Several important caveats should be considered when considering the results presented here. We labeled Nanog and Sox2 loci using knock-in fluorophore derivatives of eGFP (protein t_1/2_∼22 h), a strategy used extensively in prior studies at the Nanog locus (Chakraborty et al., 2020; Chambers et al., 2007; Faddah et al., 2013; Filipczyk et al., 2015). These reporters are best thought of as reading out a cell state, and of temporally averaged signal for Nanog and Sox2 (estimated protein t_1/2_ 2–3 hr), as opposed to reading out levels of gene expression in real time. Nanog and Sox2 are extensively regulated by other transcription factors that bind their regulatory regions, making these genes centers of a larger regulatory network determining cell state. Thus, Nanog levels fluctuate over a small range on the time scale of hours, but also fluctuate over a large range over the timescale of days, as ESCs transition between global Nanog-high and Nanog-low states (Singer et al., 2014). Therefore, it is likely the reporters used here capture global changes in state but do not capture smaller fluctuations in Nanog or Sox2 levels and likely underestimate the dynamics of cells leaving State 1 (high Nanog and high Sox2). Despite this limitation, we still detected lineages with relatively high transition rates between States 1 and 3, which correlated with differentiation potential under retinoic acid treatment.

An additional caveat concerns the technical measurement of States 1–3. We discretized and binned cells into three states and sampled them using extreme gates. While this gave us defined states to measure and allowed us to cleanly separate populations, it does mean that intermediate states were not sampled and the population was incompletely measured. The cells discarded during culture splitting in between the six-day sampling intervals were another source of incomplete measurement. Incomplete measurement could impact the measurement of Markov vs non-Markov dynamics, which we attempted to minimize by analyzing lineages detected in the extreme gates at all time points. Additionally, we calculated dynamics by rounding to whole cell numbers and proportions (Note), and while we cannot exclude an impact of rounding error on the precise dynamics calculated, we detected non-Markov behavior in a high enough fraction of lineages to feel confident in the qualitative results presented here.

The idea that kin related cells transition between states in a correlated fashion suggests the presence of as yet unknown hidden variables that may govern these transitions. We defined state in this study using Nanog and Sox2 reporters, and while our previous observations suggest these genes capture the greatest component of heterogeneity in ESCs (Chakraborty et al., 2020), this is still a relatively limited two-dimensional reduction of gene expression space. Our data suggest there are at least two additional microstates in this system, as we detected skew of lineages toward either neuroectoderm or extraembryonic endoderm upon differentiation but could not predict which lineages would go either direction. Perhaps defining the full transcriptome of ESCs along with lineage information will allow better prediction of future states. On the other hand, a recent study of hematopoiesis *in vitro* and *in vivo* captured both full transcriptomic information and lineage for single cells, yet found sister progenitors to have intrinsic fate biases that could not be accounted for by the transcriptome (Weinreb et al., 2020). Another study examined ESC differentiation into neural lineages using defined signals from the culture medium, positing a chain of unobserved molecular states that cells may transit in a non-Markov process (Stumpf et al., 2017). Our results would support this model, adding that lineage is a key variable predicting dynamics as cells expand and that non-Markov behavior can also be observed in the absence of external differentiation signals, as cells reversibly explore microstates (such as States 1–3). Many elegant systems of encoding kinship relations exist in biology, and understanding how ESCs may use such systems alongside developing a more complete picture of how cells encode their histories will be of great interest.

The results here may also have implications for cellular competition. Development in the mammalian body is known to occur in part through competition, whereby the fittest precursor cells survive and make greater contributions to the adult organism (Claveria et al., 2013; Dejosez et al., 2013). Greater fitness in cell competition experiments has often been attributed to the ability of elite clonal lineages to rapidly divide, thereby increasing their number (Shakiba et al., 2019). We find a subset of ESC lineages shows consistently high motility in transitioning between states but does not appreciably increase its growth rate and thus does not dominate the population. This may indicate that in some contexts highly dynamic lineages are those with a greater ability to switch between states in a manner distinct from elite growth ability.

Fluctuations between states in biological systems have previously been proposed to arise in part from slow global fluctuation of the transcriptome, possibly over timescales as long as a week (Huang, 2009). The results presented here would support such a model. While the source of such fluctuations is unknown, one possible source could be oscillators that may in part drive state transitions and identification of such systems will be of great interest. In mammalian embryogenesis, cells in the inner cell mass display heterogeneous levels of Nanog over a relatively small time window (E3.75–E4.75 [Shahbazi et al., 2017]), and the source of this heterogeneity remains unclear. ESCs are derived from the inner cell mass, and this enables Nanog and Sox2 state transitions to be studied in a large number of cells over several days. Since ESCs *in vivo* eventually constitute the entirety of the adult organism, understanding whether they display non-Markov behavior in a way that influences their future lineage choices during mammalian development will be of great interest. This could reveal new understandings of the true interchangeability of ESCs and their descendant cells during embryogenesis and may help identify the fluctuations giving rise to heterogeneity in key developmental factors. Together, the results presented here showing ESC state transitions are non-Markovian for some lineages may have implications for understanding the flow of biological information during state transitions and may suggest homeostasis and biological robustness are best understood both at the level of individual cells and at the level of the lineages from which they are descended.

### Limitations of the study

Several limitations apply to the present work. We measured cell state using Nanog and Sox2 reporters, which represent a limited two-dimensional view of gene expression space. Reporter half-lives were chosen to match global gene expression states, but likely underestimated dynamics of Nanog and Sox2 themselves. Additionally, states were measured using discretization, binning, and extreme gates that helped ensure purity, but came at the expense of incomplete measurement and possible blur in the estimate of dynamics. Finally, the study is limited in that no molecular mechanism for the long timescale memory was discerned. Aspects related to these limitations are discussed further above.

## STAR★Methods

### Key resources table

**Table:** 

REAGENT or RESOURCE	SOURCE	IDENTIFIER
**Antibodies**		

APC Rat Anti-Mouse CD24 conjugated antibody	BD Bioscience	Cat# 562349; RRID: AB_11151896

**Deposited data**		

FASTQ raw data	This paper	Bioproject: PRJNA670562
Code for data analysis	This paper	GitHub: https://github.com/SGarg-Lab/lineage-entropy

**Experimental models: Cell lines**		

V6.5 mouse embryonic stem cell line	Novus Biologicals	Cat# NBP1-41162

**Oligonucleotides**		

Primers for fluorophore tagging of pluripotency genes, see Method Details	This paper	N/A
Forward and reverse primers for lineage tracing, see Table S5	This paper	N/A
Flowcell primer for next generation sequencing, see Table S5	This paper	N/A
Primers for RT-qPCR, see Table S6	This paper	N/A

**Recombinant DNA**		

ClonTracer Barcoding Library	Addgene	Cat# 67267

### Resource availability

#### Lead contact

Further information and requests for resources and reagents should be directed to and will be fulfilled by the lead contact, Salil Garg (salilg@mit.edu).

#### Materials availability

Plasmids, constructs, and primers are available upon requests.

#### Data and code availability

The FASTQ files for this experiment are available on Sequence Read Archive (SRA) with Bioproject: PRJNA670562. Python scripts for processing FASTQ files generated by sequencing and subsequent analyses are available on GitHub (https://github.com/SGarg-Lab/lineage-entropy).

Any additional information required to reanalyze the data reported in this work paper is available from the Lead Contact upon request.

### Experimental model and subject details

#### Cell line information

Two mouse embryonic stem cell (ESC) lines were used in this study: V6.5 (gift from the Jaenisch Laboratory, Whitehead Institute, MIT) and V6.5 derived *Nanog-GFP/Sox2-mCerulean* generated as described below using stably-integrated DNA barcodes ((Bhang et al., 2015), Addgene #67267).

#### Cell line maintenance

Cells were cultured in liquid medium on 10-cm tissue plates pre-coated with 0.2% gelatin in phosphate-buffered saline (PBS). Cells were grown in an incubator at 37°C with 5% CO_2_. Cells were tested for mycoplasma infection every 6 months.

The liquid medium contains 415 mL of Dulbecco’s Modified Eagle Media (DMEM, Catalog number 1195073, Gibco), 5 mL of 0.1 mM L-glutamine, 5 mL of 0.1 μM non-essential amino acids, 5 mL of 0.1 μM penicillin-streptomycin antibiotics solution, 5 mL of 1 M HEPES buffer, 4 μL of 14.3 M beta-mercaptoethanol, 82.5 mL HyClone fetal bovine serum (FBS), and 55 μL of 1000U/mL Leukemia Inhibitory Factor (LIF). DMEM + additive components were filtered sterilized using a 0.45 micron filter (Catalog number 430770, Corning) before adding FBS and LIF.

### Method details

#### Fluorophore tagging of pluripotency genes

Endogenous *Nanog* and *Sox2* genes were tagged by *GFP* and *mCerulean* via CRISPR-Cas9 induced homology directed repair (HDR). Single-guided RNAs targeting upstream of the start codon (*Nanog*) or downstream of the stop codon (*Sox2*). The single-guide RNA sequence (For *Nanog* locus: 5’-CACCGTCAGTGTGATGGCGAGGGA-3’ and its complementary 3’-AAACTCCCTCGCCATCACACTGAC-5’; For *Sox2* locus: 5’-CACCGATTGGGAGGGGTGCAAAAAG-3’ and its complementary 3’-AAACCTTTTTGCACCCCTCCCAATC-5’) was cloned into PX330 plasmid using BbsI restriction site. The plasmid was then introduced to the cell using cationic lipid transfection (Lipofectamine 2000, Invitrogen, Catalog number #11668019) along with a homology-directed repair construct encoding the relevant fluorophore (*GFP* for *Nanog* and *mCerulean* for *Sox2*), T2A/P2A post-translational cleavage sequences, and a drug resistance gene (*Puromycin*^*R*^ for *Nanog* and *Blasticidin*^*R*^ for *Sox2*). Cells were then selected in culture medium with Puromycin and Blasticidin at concentrations of 2 μg/mL and 4 μg/mL, respectively, for 14 days.

#### Molecular barcoding of ESC

ESCs were labeled using ClonTracer library ((Bhang et al., 2015), Addgene #67267). *Nanog-GFP*; *Sox2-mCerulean* V6.5 ESCs were transduced with the aforementioned barcoding library by spinoculation. 101,703 transduced cells (Table S1) were selected by the expression of RFP using flow cytometric sorting. Successfully transduced cells were then cultured for ∼2 weeks until at least 10^8^ cells were present in the population. These cells were then cultured and sorted as described in Figure 1B.

#### Retinoic acid-induced differentiation of ESC

ESCs were seeded in the aforementioned liquid medium on 10-cm tissue plates pre-coated with 0.2% gelatin in phosphate buffer saline (PBS) at the density of 250,000 cells. The next day, we changed the old medium with the new N2B27 medium with 0.25 uM retinoic acid solution. N2B27 medium contained 2.5 ml of 200 mM L-glutamine (Catalog number 25030140, Gibco), 2.5 ml of 100X N2 supplement solution (Catalog number 17502048, Gibco), 5 ml of 50X B27 supplement solution (Catalog number 17504044, Gibco), 247.5 ml of Dulbecco's Modified Eagle Medium: Nutrient Mixture F-12 Ham (DMEM/F12, Catalog number D6421, Sigma), 245 ml of Neurobasal medium (Catalog number 21103049, Gibco), and 500 ul of 100 mM beta-mercaptoethanol. The 25 mM retinoic acid stock solution was made by resuspending 50 mg of retinoic acid (RA, Catalog number R2625-50MG, Sigma) in 6.67 ml of dimethyl sulfoxide (DMSO, Catalog number 472301-100ML, Sigma). Differentiating cells were cultured in this N2B27 medium with retinoic acid for 4 days, and the medium was refreshed after 48 hours.

#### Staining CD24 proteins on differentiated ESC

RA-induced differentiated ESCs could be classified into two main populations: one that expressed high CD24 marker (neuroectoderm) and low CD24 marker (extraembryonic endoderm). To quantify the amount of this protein marker, ESCs were washed with phosphate-buffered saline (PBS), harvested by trypsinization, and adjusted so their concentration was 5,000,000 cells/ml in FACS buffer (10% Hyclone Fetal Bovine Serum (FBS) in PBS). 100 μl of cell suspension was then added to wells of 96-well plates. Then 1 μg of APC Rat Anti-Mouse CD24 conjugated antibody (Catalog number 562349, BD bioscience) was added to each well before incubating at 4^o^C for an hour. Stained cells were then washed three times with described FACS buffer, before keeping at 4^o^C until analysis.

#### Reverse transcription quantitative real-time PCR (RT-qPCR) to measure gene expression differences on differentiated ESC

After being stained with APC Rat Anti-Mouse CD24 conjugated antibody for an hour, differentiated ESC were sorted into two populations, CD24^high^ and CD24^low^. RNA was then extracted from these samples by TRIzol extraction (TRIzol, Catalog number 15596018, Thermo Fisher) using the protocol from Thermo Fisher instruction manual. cDNA was synthesized using SuperScript IV Reverse Transcriptase (Catalog number 18090010, Thermo Fisher). cDNA was then amplified by different pairs of primers that target 7 different genes using SYBR green master mix. Primer sequences are given in Table S6.

#### Flow cytometry and fluorescence activated cell sorting (FACS)

Barcoded *Nanog-GFP*; *Sox2-mCerulean* V6.5 ESCs were analyzed for their Nanog-Sox2 expression on a BD LSRII HTS-2 with FACSDiva v8.0 acquisition software. Data gathered from flow cytometry were then analyzed by FlowJo V9.9. Live cells were first selected based on the forward scatter area (FSC) vs. side scatter area (SSC). Single cells were then selected based on forward scatter height (FSC-H) vs. forward scatter width (FSC-W). The expression of GFP and mCerulean (a proxy for Nanog and Sox2 expression, respectively) were observed using FITC and Pacific Blue detector channels using wild type V6.5 or singly GFP/mCerulean labeled ESC as compensation controls. For the experiment detecting ESC lineages, this barcoded fluorophore-tagged ESC line was sorted by a BD FACS ARIA machine into 3 states based on the level of GFP (representing Nanog level) and mCerulean (representing Sox2 level). Distinct states were sorted into culture medium, spun at 223 rcf for 5 minutes, and cell pellets frozen for analysis.

ESCs that were differentiated by retinoic acid for four days were analyzed for their CD24 expression on a BD LSRII HTS-2 with FACSDiva v8.0 acquisition software. Data gathered from flow cytometry were then analyzed by FlowJo V9.9. Live cells were first selected based on the forward scatter area (FSC) vs. side scatter area (SSC). Single cells were then selected based on forward scatter height (FSC-H) vs. forward scatter width (FSC-W). The expression of CD24 was observed using APC detector channels using wild type V6.5 and unstained differentiated ESCs as compensation controls. Differentiated cells were then sorted by a BD FACS ARIA machine into 2 states based on the level of CD24 (CD24^high^ and CD24^low^). Distinct states were sorted into culture medium, spun at 223 rcf for 5 minutes, and cell pellets for frozen for analysis.

FACS analyzers and sorters utilized were provided by The Swanson Biotechnology Center Flow Cytometry Facility, Koch Institute for Integrative Cancer Research at MIT.

#### Extracting DNA barcodes from ESC genome

Genomic contents from sorted cells were extracted using Sigma GenElute Mammalian Genomic DNA Prep Kit (Catalog number #G1N70) and PCR amplified using the primers listed in Table S5. DNA samples from different states and different timepoints were amplified using unique reverse primers. PCR reactions contained 10 μL of 5X NEB Phusion High-Fidelity buffer (Catalog number B0518S), 1.5 μL of DMSO, 1 μL of dNTPs (NEB, catalog number N0447S), 1 μL of 10 μM forward primer, 1 μL of 10 μM reverse primer, 0.5 μL of NEB 2000U/mL Phusion High-Fidelity DNA Polymerase (Catalog number M0530S), genomic DNA, and ddH_2_0 to 30 μL. Reactions were amplified using an annealing temperature of 55.5ºC for 25 cycles. Amplicons were pooled together for DNA next-generation sequencing.

#### Illumina sequencing

Amplicons were sequenced using Illumina MiSeq and NextSeq500 sequencers. This sequencing service was provided by MIT BioMicro Center, MIT Department of Biology. The flowcell primer 5’-CCGAGATCTACACACTGACTGCAGTCTGAGTCTGACAG-3’ was used.

#### Quality metrics for sequencing reads

Sequencing reads in FASTQ files generated from DNA next-generation sequencing were quality filtered by Phred Score (Phred +33, Illumina 1.9) and for sequencing errors compared to the reference amplicon. The reference sequence is: 5’-NNNNNNNNNNNNNNNNNNNNNNNNNNNNNNAGCAGAGCTACGCACTCTATGCTAGTGCTAGAGATCGGAAGAGCACACGTCTGAACTCCAGTCACXXXXXXXXXXATCTCGTATGCCGTCTTCTGCTTG-3’ where N represents barcodes and X sample indices, respectively. Before use in subsequent analyses, each read must pass the following criteria: 1. The sum of the Phred quality values of the barcoding region (the first 30 bases) must be greater than 80% of maximum Phred quality values (40 Phred score/base ∗ 30 base = 1200 maximum Phred score); 2. The Hamming distance (the number of base mismatches) between the read and the reference sequence in the first constant region (the 31^st^ to 95^th^ nucleotide) must be less than 6; 3. The Hamming distance between the read and the reference sequence in the second constant region (the 106^th^ to 130^th^ nucleotide) must be less than 3; 4. The Hamming distance between the read’s sample index and one of the sample indices must be less than 2. The number of reads that passed these quality metrics are listed in Table S4. Reads that passed the quality metrics were then separated based on sample indices. (Note that each sample was amplified by a unique reverse primer that contained a distinct sample index. This allowed us to separate them in this pipeline.) Finally, reads that passed the quality standard with barcodes less than 6 Hamming distance away from each other were collapsed together.

#### Total count normalization method

Quality filtered reads were then normalized into cell counts based on the State sample from which they originated. Specifically, reads that were in State 1 were normalized to 90,000,000 cells whereas State 2 and State 3 reads were normalized to 5,000,000 cells. This ratio of State 1: State 2: State 3 = 90:5:5 was approximated from the distribution of cells in the FACS plot (Figures 1A and S2B) and normalized to the total of 10^8^ cells sorted. Please note that this calculation approach allows samples with different sequencing depth to normalize to the same total number of cells if they represent cells in the same State. For example, all State 1 cells from Day 0, 6, 12, 18, and 24 are always normalized to 90,000,000 cells. Lineages are further considered if and only if the sum of their read numbers in State 1, State 2, and State 3 is greater than 0 at all timepoints.

Reads from CD24^high^ and CD24^low^ samples were normalized to 3,000,000 and 1,000,000 cells, respectively, since these are the actual number of cells sorted in each sample.

#### Motility of ESC lineages

Relative transition amount or motility is defined by the Cartesian distance between the location of a lineage in the first timepoint (x_1_, y_1_) to the location in the second timepoint (x_2_, y_2_) on the ternary plot. In other words,

$$Motility=\sqrt{{\left(x_{1}-x_{2}\right)}^{2}+{\left(y_{1}-y_{2}\right)}^{2}}$$

Using ternary plot coordinates for (x,y) where a lineage entirely in State 1 is (1,0), State 2 is (0,1), and State 3 is (0,0). Note that the motility is always between 0 (no change in state proportions between two timepoints) and $\sqrt{2}$ (100% change along State 1-State 3 axis). The maximum change of state proportions along State 1-State 2 axis and State 2-State 3 axis is 1. This weighting was regarded as appropriate given the greater gene expression differences between States 1 and 3 as compared to State 2 (Figure 1A and (Chakraborty et al., 2020). Lineages were ranked (Figure 5A) and separated into 10 groups based on their **overall motility** (sum of motilities in all 4 transitions). The motility in each transition of each group was shown in Figures 5C, 5D, S8, and S9. Percent Motility (used in Figures 5, S8, and S9) was calculated by:

**Percent Motility** = (Motility of that lineage /$\sqrt{2}$) ∗ 100

#### Calculating the proportion of lineages in various states over time

To simplify our analysis, each lineage was assigned to one of the three bins – State 1 bin, State 2 bin, and State 3 bin – based on which state had the highest number of cells (plurality vote). For example, lineage which had 1000 State 1 cells, 500 State 2 cells, and 500 State 3 cells, would be assigned to State 1 bin. Using this method, we separated our 2,560 analyzable lineages into 2,323 State 1 lineages, 86 State 2 lineages, and 151 State 3 lineages, and we continued to follow how lineages in different bins changed their state assignment over time from Day 0 to Day 24 (Figure S7). The decision tree was produced by Python networkx module. The width of edges corresponded to the proportion of lineages in that transition. Different shades of gray represented different timepoints.

#### Calculating the correlation of motility among replicate experiments

To determine whether the correlation in motility between two experimental replicates was significant (Figures S10C and S10D), we calculated a correlation of **randomized motility** using the following method: 1. Identify lineages that exist in both replicates; 2. For each replicate (WT1 and WT2), collect the experimental motility values of each transition for each lineage; 3. Randomly assigned motility values to each lineage from the distribution of motilities within that replicate using the data collected from step (2); 4. Calculate log_10_ of percent motilities from these randomly assigned WT1 and WT2 motility values; 5. For each lineage in WT1 and WT2 replicates, plot the data and calculate Pearson correlation coefficients (r-values) for each transition. The comparison between empirically observed motility correlation (Figure S10C) and those randomly selected (Figure S10D) is shown. Upon 100,000 trials of this randomization procedure, R^2^ between randomly assigned WT1 and WT2 motilities ranged from 0 to 0.01 for these trials.

#### Fisher’s exact test of independence between different state histories

To determine whether the difference in state distributions between two different state histories was significant (Table S2, “Motif Analysis” sheet; Table S3, “Motif Analysis” sheet), we compared the number of lineages in each state at Day 24 for lineages with two distinct histories for lineages that occupied the same state on Day 18 using Fisher’s Exact Test. We restricted analysis to histories that contained lineages occupying all states in the last timepoint such that the Fisher statistic is defined. Benjamini-Hochberg false discovery rate was used for multiple testing correction. The contingency tables and the corrected p-values of *Nanog-Sox2* state transitions are found in Table S2, “Fisher exact test” sheet. The contingency tables, p-values, and the Bonferroni corrected alpha value of *motility* state transitions are found in Table S3, “Fisher exact test” sheet.

#### Note

##### Estimating overall transitional probability matrix from FACS information

First, we want to understand the how cells in three different states (State 1: High Nanog/High Sox2; State 2: Low Nanog/High Sox2; State 3: Low Nanog/Low Sox2) interconvert between one another in the bulk population. We model this system using Markovian model in which the cell state percentages **after** cell state transition (let’s called this matrix Y, values always between 0 and 100) can be accurately predicted by cell state percentages before cell state transition (matrix X, values between 0 and 100) multiplied by a transitional matrix M.

XM=Y

We use percentages of cells in different FACS data (Figure S2B) in X and Y matrices. The pre-transition matrix X is a 4×3 matrix, where rows contain the proportions of cells in State 1, State 2, State 3 on Day 0, Day 6, Day 12, and Day 18. Its counterpart, the post-transition matrix Y is also a 4×3 matrix, where rows contain the proportions of cells in State 1, State 2, State 3 on Day 6, Day 12, Day 18, and Day 24. The unknown transitional matrix M is a 3×3 matrix that explains the state transition between X and Y.

To solve for a matrix M, we can use ordinary least squares (OLS) estimation to calculate the matrix by minimizing the sum of squared errors. The error of estimation is therefore given by r=Y−XM where r is a 4 × 3 matrix. We calculate the sum of squared errors (noted as S) by multiplication of r with $r^{\text{T}}$, where $r^{\text{T}}$ denotes the transpose of r.

$$S=\left\langle r,r\right\rangle =rr^{\text{T}}=\left(Y-XM\right){\left(Y-XM\right)}^{\text{T}}=\left(Y-XM\right)\left(Y^{\text{T}}-M^{\text{T}}X^{\text{T}}\right)=YY^{\text{T}}-YM^{\text{T}}X^{\text{T}}-XMY^{\text{T}}+XMM^{\text{T}}X^{\text{T}}$$

A matrix M that minimize the sum of squared errors S must occur at the critical point where the gradient of S with respect to M equals 0.

$$\frac{\partial S}{\partial M}=\frac{\partial \left({\text{YY}}^{\text{T}}-{\text{YM}}^{\text{T}}{\text{X}}^{\text{T}}-{\text{XMY}}^{\text{T}}+{\text{XMM}}^{\text{T}}{\text{X}}^{\text{T}}\right)}{\partial \text{M}}=-2{\text{X}}^{\text{T}}\left(\text{Y}-\text{XM}\right)={\text{X}}^{\text{T}}\text{Y}-{\text{X}}^{\text{T}}\text{XM}=0$$

Therefore,

$$\begin{array}{c} \text{M}={\left({\text{X}}^{\text{T}}\text{X}\right)}^{-1}{\text{X}}^{\text{T}}\text{Y} \end{array}$$

Please note that matrix M is not necessarily right stochastic since we used unbounded linear least square estimation, and we do not normalize this matrix before performing downstream calculation.

We can use this calculated matrix M to estimate the state proportions after transitions (let’s called this matrix $X^{\prime }$) by performing the following calculation

$$X^{\prime }=XM$$

Then we can perform row-wise chi-squared comparison between $X^{\prime }$ (cell state percentages under Markovian assumptions) and Y (observed cell state percentages after transition), to check if matrix M accurately predicted the distribution of cells. If the p-values from chi-square test are greater than the alpha value (0.05), which in this case, they are, it means that matrix M can predict the distribution after the transition process, and hence cell state transitions in the bulk population is Markovian.

#### Estimating overall transitional probability matrix using lineage information

We can model the system of three cell states where cells in each state can either stay in the same state or convert to one of the other two states after each transition. In this section, we describe how we estimate the transitional probabilities that explain how lineages change states between timepoints.

Each lineage contains information about the proportion of cells in different states, which is known for all timepoints (Day 0, Day 6, Day 12, Day 18 and Day 24). We will focus on two contiguous timepoints for clarity (note this will generalize to any transition): the data from Day 0 and Day 6.

Let $X_{i}$ and $\;Y_{i}$ be 1×3 row vectors that contain the proportion of 3 states of lineage i on Day 0 and Day 6, respectively. In other words,

$$\begin{array}{c} {\text{X}}_{\text{i}}=\left[\begin{array}{lll} {\text{P}}_{\text{s}1\text{,i}} & {\text{P}}_{\text{s}2\text{,i}} & {\text{P}}_{\text{s}3\text{,i}} \end{array}\right]\\ {\text{Y}}_{\text{i}}=\left[\begin{array}{lll} {\text{P}}_{\text{s}1\text{,i}}^{\prime } & {\text{P}}_{\text{s}2\text{,i}}^{\prime } & {\text{P}}_{\text{s}3\text{,i}}^{\prime } \end{array}\right] \end{array}$$

Where for example ${\text{P}}_{\text{s}2,\text{i}}$ indicates the proportion of lineage i that is in State 2 at timepoint Day 0. Therefore, the row sums of $X_{i}$ and $Y_{i}$ will always equal to 1.

Because these three states can interconvert between one another, there must be a matrix M of transitional probabilities can explain the conversion from $X_{\text{i}}$ to $Y_{\text{i}}$. Therefore, we can write,

$$\begin{array}{c} X_{\text{i}}M=Y_{\text{i}} \end{array}$$

$$\begin{array}{c} \text{where,}\;M=\left[\begin{array}{lll} {\text{M}}_{11} & {\text{M}}_{12} & {\text{M}}_{13}\\ {\text{M}}_{21} & {\text{M}}_{22} & {\text{M}}_{23}\\ {\text{M}}_{31} & {\text{M}}_{32} & {\text{M}}_{33} \end{array}\right] \end{array}$$

Please note that the multiplication of $X_{\text{i}}$ and M describes how state proportions $\left[\begin{array}{lll} {\text{P}}_{\text{s}1,\text{i}} & {\text{P}}_{\text{s}2,\text{i}} & {\text{P}}_{\text{s}3\text{,i}} \end{array}\right]$ change to $\left[\begin{array}{lll} {\text{P}}_{\text{s}1\text{,i}}^{\prime } & {\text{P}}_{\text{s}2,\text{i}}^{\prime } & {\text{P}}_{\text{s}3,\text{i}}^{\prime } \end{array}\right]$. And the M calculated in **this section is different** from M estimated in the previous section, **Estimating overall transitional probability matrix from FACS information.**

Here, we want to solve a system of linear equations for matrix M. However, considering only one lineage leaves 3 constraints with 9 unknowns, making it difficult to explicitly consider M for any single lineage. Hence, we utilize the 2,560 lineages that exist in all timepoints (Figure 1D). We can use this information to estimate the transitional probability matrix M that explains the transitions between states for **all** lineages on averageof I between Day 0 to Day 6.

This now gives two 2560×3 matrices instead of two 1×3 row vectors. We note the matrix of state proportions of all lineages on Day 0 as X and its Day 6 counterpart as Y. Note that row j of X and Y represents the state proportions of lineage j on Day 0 and Day 6, respectively. From this, we can write,

$$\begin{array}{c} XM=Y \end{array}$$

and can solve for the matrix M that minimizes the error of transforming X to Y. Because this is a system of linear equations, we use ordinary least squares (OLS) estimation to calculate the matrix M by minimizing the sum of squared errors, explained in **Estimating overall transitional probability matrix from FACS information**.

$$\begin{array}{c} M={\left(X^{\text{T}}X\right)}^{-1}X^{\text{T}}Y \end{array}$$

Using the knowledge from this derivation, we can estimate the transitional probability matrix M that explains the transition from one timepoint to another subsequent timepoint by substituting X as a matrix of state proportions in an earlier timepoint and Y as a matrix of state proportions in a later timepoint.

In order to find an average transitional probability matrix across all transitions, instead of using 2560×3 X and Y matrices, we combine the state proportions of all lineages on Day 0, Day 6, Day 12 and Day 18 into a 10240×3 matrix $X^{\prime }$ and combine the state proportions of all lineages on Day 6, Day 12, Day 18, and Day 24 into a 10240×3matrix $Y^{\prime }$. Note that row j of both $X^{\prime }$ and $Y^{\prime }$ represents the state proportions of the same lineage; $X\prime_{\text{j}}$ has the information from the earlier timepoint while $Y\prime_{\text{j}}$ has the information from the later timepoint.

Because there are 10240 rows in $X^{\prime }$ and, $Y^{\prime }$ the change in extreme state proportions (i.e., $\left[\begin{array}{ccc} 0 & 0 & 1 \end{array}\right]$ to $\left[\begin{array}{ccc} 1 & 0 & 0 \end{array}\right]$) do not greatly affect the least-square estimated matrix M. And the resulting matrix M is **right stochastic** (i.e., each row sums to 1, and the value of each entry is always between 0 and 1). Therefore, further normalization is not needed.

#### Estimating lineage-specific transitional probability matrix

To calculate a transitional probability matrix that is specific to each unique lineage i, we use two 4×3 matrices, $X_{\text{i}}$ and $Y_{\text{i}}$. Rows in matrix $X_{\text{i}}$ represent the proportions of cells from lineage i in three states on Day 0, Day 6, Day 12, and Day 18, respectively. On the other hand, row in matrix $Y_{\text{i}}$ represents the proportions of cells on Day 6, Day 12, Day 18, and Day 24. Assume that there is a matrix $M_{\text{i}}$ that can explain the transition between cell state proportions in the earlier timepoints ($X_{\text{i}}$) to the later timepoints ($Y_{\text{i}}$), we can write

$$\begin{array}{c} X_{\text{i}}M_{\text{i}}=\;Y_{\text{i}} \end{array}$$

Note that the row sums of $X_{\text{i}}$ and $Y_{\text{i}}$ will always equal to 1.

Then we can use least square estimation to find the $M_{\text{i}}$ that best describe this set of linear questions.

$$M_{\text{i}}={\left(X_{\text{i}}^{\text{T}}X_{\text{i}}\right)}^{-1}X_{\text{i}}^{\text{T}}Y_{\text{i}}$$

Therefore, we have 2,560 unique transitional probability matrices $M_{\text{i}}$, which describe how each specific lineage change their state.

However, unlike the overall transitional probability matrix M which is calculated from 10240×3 matrices, $X^{\prime }$and $Y^{\prime }$, the lineage-specific transitional probability matrix $\;M_{\text{i}}$ is calculated from 4×3 matrices, $X_{\text{i}}$ and $Y_{\text{i}}.$ We use this matrix $M_{\text{i}}$ in downstream analyses without further normalization.

#### Determining the Markovian property of each transition across all lineages

A transition is Markovian if the lineage-specific transitional matrix (described in previous section) can predict the distribution of cells across three states on the later timepoint without statistical significance in differences between expected and observed values. For example, a transition from Day 0 to Day 6 of lineage i is Markovian if the distribution of cell states on Day 6 can be predicted by $M_{\text{i}}$.

To evaluate the Markovian property of lineage i at the transition between Day a to Day b, we normalize cells in each timepoint to 1,000,000 cells instead of 100,000,000 cells to reflect the real number of sorted cells (Table S1). This change in normalization makes several lineages become smaller and round down to less than 1 cell at some timepoint (i.e., some timepoints now have 0 cells), reducing the total number of analyzable lineages from 2,560 to 1,736.

Let $N_{\text{i}}^{\text{b}}$, as 1×3 matrix that describes the distribution of **cell numbers** in three states on Day b. And let’s define $X_{\text{i}}^{\text{a}}$ and $X_{\text{i}}^{\text{b}}$ as 1×3 matrices that represent the distribution of **cell proportions** among three states on Day a and Day b, respectively.

Note that each entry in $N_{\text{i}}^{\text{b}}$ is a positive integer describing the number of cells in each state, while each entry of $X_{\text{i}}^{\text{a}}$ and $\;X_{\text{i}}^{\text{b}}$ is some real number between 0 and 1 showing the fraction of cells occupying a particular state.

Next, to check if a transition matrix $\;M_{\text{i}}$ can predict the empirical distribution, we find the **expected** distribution of cells of Day b from Day a, ${X^{\prime }}_{\text{i}}^{\text{b}}$, by calculating

$${X^{\prime }}_{\text{i}}^{\text{b}}=X_{\text{i}}^{\text{a}}M_{\text{i}}$$

Then we can calculate the expected distribution of cells on Day b, ${N^{\prime }}_{\text{i}}^{\text{b}}$

$${N^{\prime }}_{\text{i}}^{\text{b}}=\;{X^{\prime }}_{\text{i}}^{\text{b}}\cdot \sum N_{\text{i}}^{\text{b}}$$

where $\sum N_{\text{i}}^{\text{b}}\;$is the sum of the number of cells across three states on Day b; in other words, it is the total number of cells on Dayb.

Now we can test whether $\;N_{\text{i}}^{\text{b}}$ (the expected distribution of cells on Day b calculated by the Markovian assumption) and ${N^{\prime }}_{\text{i}}^{\text{b}}$ (the observed distribution of cells on Day b) are  b significantly different using chi-square test of homogeneity using the entries from $\;N_{\text{i}}^{\text{b}}$ as observed values and the entries from $\;N\prime_{\text{i}}^{\text{b}}$ as expected values. Note that because lineage-specific transition matrix $\;M_{\text{i}}$ may have some negative values, we remove those negative or zero estimated cell number from the analysis, and thus refer to them as uninformative lineages. This chi-square homogeneity test only uses positive values from both $N_{\text{i}}^{\text{b}}$ and ${N^{\prime }}_{\text{i}}^{\text{b}}$.

P-values from these tests across 1,736 lineages during transition (a,b) are then corrected using Benjamini-Hochberg false discovery rate.

#### Estimating lineage-specific transitional probability matrix of all non-Markovian lineages

After understanding the Markovian property in each lineage, we identified 114 lineages where all 4 state transitions are non-Markovian. We sought to understand the distribution of values in their transitional probability matrices. Let $X_{\text{i}}$ be a 4×3 matrix representing the proportions of cells in three states on Day 0, 6, 12, and 18, and $Y_{\text{i}}$ be a 4×3 matrix representing the proportions of cells in three states on Day 6, 12, 18, and 24. Assume that there is a matrix $M_{\text{i}}$ that can explain the transition between cell state proportions in the earlier timepoints ($X_{\text{i}}$) to the later timepoints ($Y_{\text{i}}$), we can write

$$\begin{array}{c} X_{\text{i}}M_{\text{i}}=\;Y_{\text{i}} \end{array}$$

Note that the row sums of $X_{\text{i}}$ and $Y_{\text{i}}$ will always equal to 1.

We used scipy module scipy.optimize.lsq_linear to estimate the lineage-specific transitional probability $M_{\text{i}}$. Please note that we have added a linear constraint to make sure the sum of each row in $M_{\text{i}}$ will be 1 and each $M_{\text{i}}$ will be right stochastic. The distributions of all values in 114 transitional probability matrices are shown in Figure S4C.

#### Estimating growth-birth-death rate

We next sought to use our knowledge of transitional probabilities in the system of ESC lineages to estimate the net rate of growth, birth, and death events for mouse embryonic stem cells making all types of state transitions. Calculating the net growth-birth-death rate is not trivial because this rate is intertwined with state transition rates calculated in **Estimating overall transitional probability matrix.** In this section, we focus on extracting the net growth-birth-death rate once average rates of transition between states are taken into account. Please note that the calculation of this growth-birth-death rate is not used in any Markovian analysis. It is calculated to understand the change in cell size for cells undergoing different state transitions.

We first find the distributions of cells in State 1, State 2, and State 3 across all lineages predicted by the transition matrix M for a given lineage making a transition between two timepoints. The number of cells of each lineage in each state at each timepoint is calculated from the empirical data as described (see STAR Methods). We consider the difference between the number of cells at the second timepoint predicted by the transition matrix M and the number of cells empirically observed in each state at this timepoint for this lineage as a result of growth, birth, and death events.

To demonstrate, we will use the data from Day 0 and Day 6 as an example, noting that this procedure generalizes to analysis of data from all other contiguous timepoints. Let $U_{\text{i}}$ and $V_{\text{i}}$ be 1×3 row vectors that contain the normalized cell number in all three states for lineage i on Day 0 and Day 6, respectively.

In other words,

$$\begin{array}{c} U_{\text{i}}=\left[\begin{array}{lll} {\text{U}}_{\text{s}1\text{,i}} & {\text{U}}_{\text{s}2\text{,i}} & {\text{U}}_{\text{s}3\text{,i}} \end{array}\right]\\ V_{\text{i}}=\left[\begin{array}{lll} {\text{V}}_{\text{s}1\text{,i}} & {\text{V}}_{\text{s}2\text{,i}} & {\text{V}}_{\text{s}3\text{,i}} \end{array}\right] \end{array}$$

The expected distribution of cells after Day 0 cells transition between states can be found by multiplying $U_{\text{i}}$ with transitional probability matrix M we calculated in **Estimating overall transitional probability matrix**. The product is $U_{\text{i}}^{\prime }$.

$$\begin{array}{c} U_{i}^{\prime }=U_{i}M \end{array}$$

Here, vector $U_{\text{i}}^{\prime }$ describes the expected number of cells on Day 6 due to the state transition alone. Vector $V_{\text{i}}$ contains the observed number of cells for lineage i on Day 6. We assume the difference between these two vectors stems from growth, birth, and death processes happening between Day 0 and Day 6 timepoints for this lineage. To model this process, we introduce a 3×3 matrix G which describes the difference between the number of cells in $U_{\text{i}}^{\prime }$ and $V_{\text{i}}$. In other words, matrix G describes the rate of change cell numbers in different states after considering the rate of transition.

Mathematically,

$$\begin{array}{c} U_{i}^{\prime }G \end{array}=V_{\text{i}}$$

Note that each entry (a,b) of the matrix G describes the amount of cell growth or cell death between state a before the transition and state b after the transition. If the value in entry (a,b) is greater than one then it implies that cells proliferate in the transition from a to b. On the other hand, if the value in entry (a,b) is lower than one then cells must reduce in their number during the transition from a to b.

However, we cannot solve for matrix G for an individual lineage because the system of linear equations has more unknowns (9) than constraints (3). Hence, we again utilize the 2,560 lineages that are present at all timepoints (Figure 1D), and use this information from all lineages to solve for G that minimizes the difference of state distributions using the ordinary least squares method.

Let U and V be 2560×3 matrices that represent the distributions of cells in three states of all 2,560 lineages on Day 0 and Day 6, respectively. We can write,

$$\begin{array}{cc} UG & =V \end{array}$$

From ordinary least square estimation,

$$\begin{array}{c} G={\left(U^{\text{T}}U\right)}^{-1}\left(U^{\text{T}}V\right) \end{array}$$

Here G is a transition matrix describing the change in cell numbers between Day 0 and Day 6. We can use this fact to find growth-birth-death transition matrices between two other contiguous timepoints.

To find the growth-birth-death rate between two timepoints, we use matrix G to find the expected number of cells in different states after the transition process.

Let a 1×3 row vector N that describes the number of cells in different states. In other word, $N=\left[\begin{array}{lll} {\text{N}}_{\text{s}1} & {\text{N}}_{\text{s}2} & {\text{N}}_{\text{s}3} \end{array}\right]$. Assuming that there are 100 cells in State 1 at the first timepoint, we want to know how these cells change their number between timepoints due to the growth-birth-death process alone. We can find the expected number of cells $E_{\text{s}1}$ in different states after cell state transitions and growth-birth-death processes by calculating

$$\begin{array}{c} N_{\text{s}1}=\left[\begin{array}{lll} 100 & 0 & 0 \end{array}\right]\\ E_{\text{s}1}=N_{\text{s}1}MG \end{array}$$

where Mis the transitional probability matrix we derived from the least square estimation calculated in **Estimating overall transitional probability matrix using lineage information** and G is the rate of cell change matrix derived above.

The rate of change in State 1 cell number due to the growth, birth, death processes is then the entry-wise division (Hadamard division) between $E_{\text{s}1}$ and $N_{\text{s}1}M$, where $E_{\text{s}1}$ represents the number of cells after the transition and growth-birth-death process, whereas $N_{\text{s}1}M$ represents the number of cells after the transition process alone. In other words, growth-birth-death rate_s1_ = $\left[\begin{array}{lll} E_{\text{s}1,1}/{\left(N_{s1}M\right)}_{1} & E_{\text{s}1,2}/{\left(N_{s1}M\right)}_{2} & E_{\text{s}1,3}/{\left(N_{s1}M\right)}_{3} \end{array}\right]$ where $\;E_{\text{s}1,\text{i}}$ is the number of cells after the transition and growth-birth-death process that originate from state 1 and convert to state and ${\left(N_{\text{s}1}M\right)}_{\text{i}}$ is the number of cells after the transition process only that originate from state 1 and convert to state i.

If the growth-birth-death rate_i,j_ is greater than 1, this implies that cells increase size during the transition from i to j, whereas a value lower than 1 means cells decrease size during that transition.

We can calculate the rate of change in the number of cells in State 2 and 3 using the method above.

To calculate an overall growth-birth-death rate across all 5 timepoints, we form a matrix $U^{\prime }$ that contains the number of cells in each state from all lineages from 4 timepoints (Day 0, Day 6, Day 12 and Day 18) and form a matrix $V^{\prime }$ that contains the number of that contains the number of cells in each state from all lineages from 4 subsequent timepoints (Day 6, Day 12, Day 18 and Day 24). Please note that entries in each row of $U^{\prime }$ and $V^{\prime }$ are from the same lineages; entries in $U^{\prime }$ are from an earlier timepoint while their counterparts in $V^{\prime }$ are from the subsequent timepoint. From this we can calculate rate of change in cell size matrix $G^{\prime }$ and the growth-birth-death rate for each state the way we have mentioned above.

Estimates for the growth-birth-death rate for each state are shown in Figure S4B. The error estimation and confidence interval are calculated from 80% bootstrapping over 100,000 iterations.

#### Calculating lineage entropy

We start here with definitions of concepts as they are considered in the present study; these may be familiar to many readers. In information theory, entropy of a variable reveals the average amount of information or uncertainty in its outcomes. Given a random variable X with n possible outcomes $x_{1},x_{2},...,x_{n}$ that occurs with probability $P\left(x_{1}\right),P\left(x_{2}\right),...,P\left(x_{n}\right)$, the informational entropy of X can be mathematically defined as

$$\begin{array}{cc} H\left(X\right) & =-\overset{n}{\underset{i=1}{\sum }}P\left(x_{i}\right)\log\;P\left(x_{i}\right) \end{array}$$

where entropy is always between 0 and 1. This quantity may be familiar to readers as the Shannon Entropy.

A further intuition can be found by considering a coin-flip thought experiment. If the coin is fair, there are two possible outcomes (heads and tails) both occuring at equal probability 1/2. Therefore, if flipping this coin, we do **not** know for sure which result will be obtained, meaning this system has maximal uncertainty and maximal information to be gained once we know the result of the coin flip, high information entropy). The entropy in this system in this case is

$$H\left(X\right)=-\overset{n}{\underset{i=1}{\sum }}P\left(x_{i}\right)\log\;P\left(x_{i}\right)=-\left(\frac{1}{2}\log\frac{1}{2}+\frac{1}{2}\log\frac{1}{2}\right)=1$$

On the other hand, if we are flipping a coin that has heads on both sides, we do know for sure that after the coin flip the result will be heads and this does not yield us any new information (minimal uncertainty, minimal informational entropy, minimal information to be gained once the result of the coin flip is known). Mathematically,

$$H\left(X\right)=-\overset{n}{\underset{i=1}{\sum }}P\left(x_{i}\right)\log\;P\left(x_{i}\right)=-\left(1\;\log\;1\right)=0$$

In general, the system with more uncertainty is considered to have more information content to be gained and higher informational entropy value.

We can use this concept to describe the lineage entropy (or informational entropy) in each lineage, consistent with the idea that lineages with more heterogeneous proportion amongst states should have higher entropy than lineages occupying only one cell state, in analogy to our coin flip.

According to the empirical data, the steady-state distribution of State 1, State 2 and State 3 given by averaging all values is at $\left[\begin{array}{lll} P\left(s1\right) & P\left(s2\right) & P\left(s3\right) \end{array}\right]=\left[\begin{array}{lll} 0.73 & 0.15 & 0.12 \end{array}\right]$. We calculate the lineage entropy $H^{\prime }(L)$ of a lineage L by:

$$\begin{array}{cc} H^{\prime }\left(L\right) & =-\left(P^{\prime }\left(s1\right){\log\;}_{3}P^{\prime }\left(s1\right)+P^{\prime }\left(s2\right){\log\;}_{3}P^{\prime }\left(s2\right)+P^{\prime }\left(s3\right){\log\;}_{3}P^{\prime }\left(s3\right)\right) \end{array}$$

where $P^{\prime }\left(s1\right),P^{\prime }\left(s2\right)$, and $P^{\prime }\left(s3\right)$ are the scaled proportion of State 1, State 2, and State 3 in lineage L respectively. In other words,

$$\begin{array}{cc} P^{\prime }\left(s1\right) & =\left\{ \begin{array}{cc} \frac{P\left(s1\right)}{0.73}\times \frac{1}{3} & 0\leq P\left(s1\right)\leq 0.73\\ 1+\frac{P\left(s1\right)-1}{1-0.73}\times \frac{2}{3} & 0.73<P\left(s1\right)\leq 1 \end{array}\right.\\ P^{\prime }\left(s2\right) & =\left\{ \begin{array}{cc} \frac{P\left(s2\right)}{0.15}\times \frac{1}{3} & 0\leq P\left(s2\right)\leq 0.15\\ 1+\frac{P\left(s2\right)-1}{1-0.15}\times \frac{2}{3} & 0.15<P\left(s2\right)\leq 1 \end{array}\right.\\ P^{\prime }\left(s3\right) & =\left\{ \begin{array}{cc} \frac{P\left(s3\right)}{0.12}\times \frac{1}{3} & 0\leq P\left(s3\right)\leq 0.12\\ 1+\frac{P\left(s3\right)-1}{1-0.12}\times \frac{2}{3} & 0.12<P\left(s3\right)\leq 1 \end{array}\right. \end{array}$$

This scaling ensures that the maximum lineage entropy occurs at the steady-state distribution instead of. $\left[\begin{array}{lll} 0.33 & 0.33 & 0.33 \end{array}\right]$

Please note that these scaled proportions are not real probabilities because they don’t add up to 1, and there is no additional normalization required for calculating $H^{\prime }\left(L\right).$

### Quantification and statistical analysis

Figure 2 evaluates the Markovian property of the system on a population level. The system is Markovian if the distribution of all cells in the system across three states can be predicted using associated transitional probability matrices. (Adjusted p-value > 0.05 from chi-square test of homogeneity). Otherwise, the system is non-Markovian. Results show that the system is Markovian in all timepoints (p > 0.05).

Figure 4B shows the pattern of state transitions of all lineages in all time points. Lineages are classified as Markovian lineages if the empirical number of cells in three states can be accurately predicted using associated transitional probability matrices. (Adjusted p-value > 0.05 from chi-square test of homogeneity and Benjamini-Hochberg correction.) On the other hand, if the matrices cannot estimate the empirical distribution of cell states on the next time point, lineages are classified as non-Markovian. (Adjusted p-value < 0.05 from chi-square test of homogeneity and Benjamini-Hochberg correction.)

Figure 5B shows the relationship between memory and motility. The relative total amount of transition is shown for all lineages, and compared to lineages with all Markovian or all non-Markovian transitions, using Kolmogorov-Smirnov test. p-value < 0.001 (∗∗∗) for all comparisons.

Figure 6C shows the ratio of CD24^high^ (neuroectoderm) to CD24^low^ (extraembryonic endoderm) among lineages with different total amount of transitions (motility). F-test for variance p < 0.01 for all deciles compared against all lineages except for the fifth decile, which was not significant.

In Figure 6D, the top 4 deciles of motility and the bottom 4 deciles of motility are grouped together and compared to the distribution of CD24^high^ (neuroectoderm) to CD24^low^ (extraembryonic endoderm) in all lineages group. F-test for variance p < 0.001 (∗∗∗) for both groups compared to all lineages.

Figure S13B compares the fold expression of different gene markers in CD24^high^ (neuroectoderm) to CD24^low^ (extraembryonic endoderm) cells. P-values for two-sample t-tests are shown. Asterisks indicate levels of significance (∗ p < 0.05, ∗∗ p < 0.01, ∗∗∗ p < 0.001).

Figure S13C shows the ratio of descendants in CD24^high^ vs CD24^low^ populations for lineages in States 1–3 across all time points. F-tests for variance were conducted comparing each state to all lineages and p-values < 0.001 (∗∗∗) are indicated.

## Supplemental information

Document S1. Figures S1–S13 and Tables S1, S4, and S6

Table S2. Probability that each lineage transitions between *Nanog-Sox*2 states over 5 timepoints, related to STAR Methods

Table S3. Probability that each lineage transitions between motility states over 5 timepoints, related to STAR Methods

Table S5. PCR primers used in amplifying DNA barcodes and next-generation sequencing, related to STAR Methods
