# Supplementary material for: Lineages of embryonic stem cells show non-Markovian state transitions
Source: iScience. 2021 Jul 17;24(8):102879. doi: 10.1016/j.isci.2021.102879 (PMC8353490; doi:10.1016/j.isci.2021.102879)
Supplement: Document S1. Figures S1–S13 and Tables S1, S4, and S6 [file mmc1.pdf]

iScience, Volume *24*

Supplemental information

Lineages of embryonic stem cells show non-Markovian state transitions

Tee Udomlumleart, Sofia Hu, and Salil Garg

A

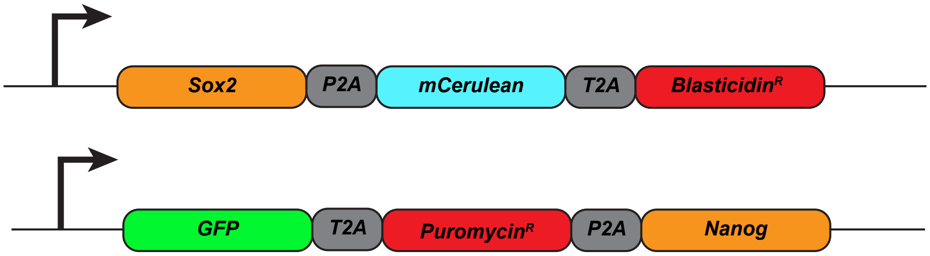
Sox2
P2A
mCerulean
T2A
Blasticidin^R
GFP
T2A
Puromycin^R
P2A
Nanog

B

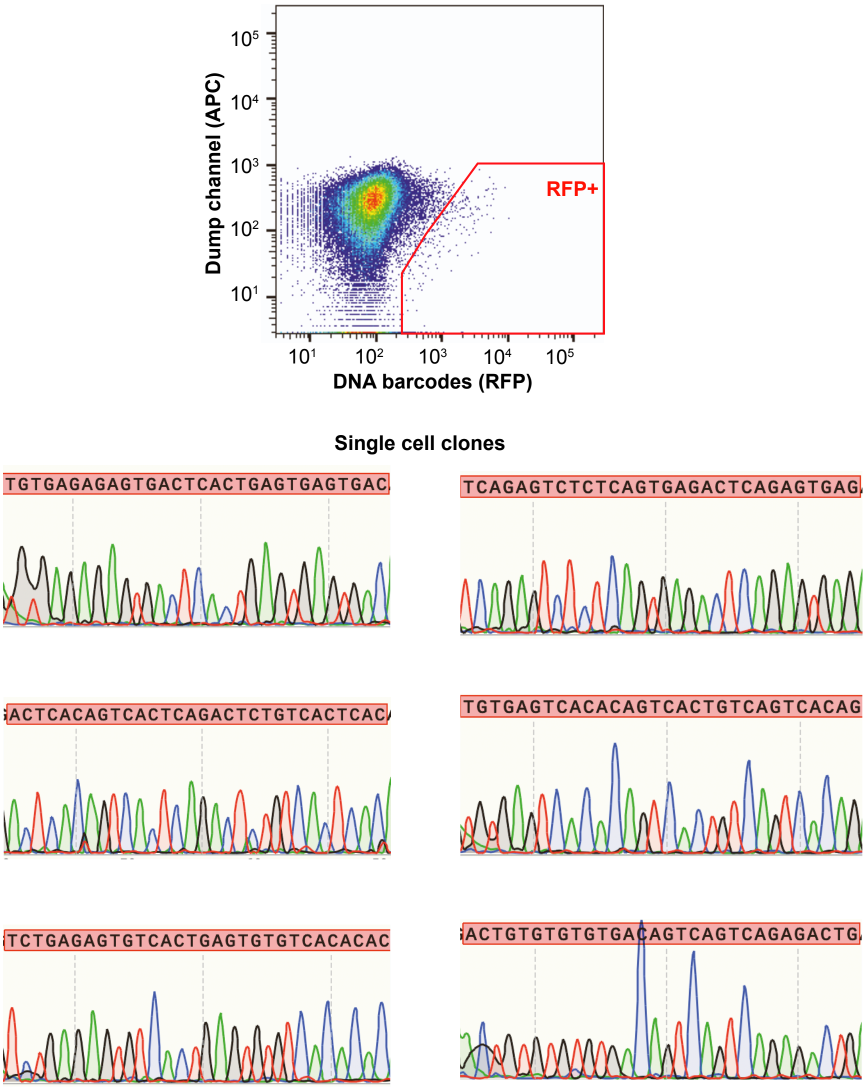
Dump channel (APC)
DNA barcodes (RFP)
RFP+
Single cell clones
TGTGAGAGAGTGACTCACTGAGTGAGTGAC
TCAGAGTCTCTCAGTGAGACTCAGAGTGAG
ACTCACAGTCACTCAGACTCTGTCACTCAC
TGTGAGTCACACAGTCACTGTCAGTCACAG
TCTGAGAGTGTCACTGAGTGTGTCACACAC
ACTGTGTGTGTGACAGTCAGTCAGAGACTG

Figure S1. ESC lineage labeling, related to Figure 1. (A) Schematic of loci for generation of *Sox2-P2A-Cerulean* and *GFP-P2A-Nanog*. *Blasticidin*^{R} and *Puromycin*^{R} refer to genes encoding resistance to the selectable markers blasticidin and puromycin, respectively. Insertions were verified heterozygous by locus PCR. **(B)** Barcoding and selection of ESC lineages using the library provided by (Bhang et al., 2015). The barcoding library encodes a red fluorescent protein (RFP) that was selected using the shown flow cytometric gate. To confirm ESC were singly barcoded, we grew single cells into cell lineages and sequenced the barcoding region using Sanger sequencing. Traces for six representative clones are shown.

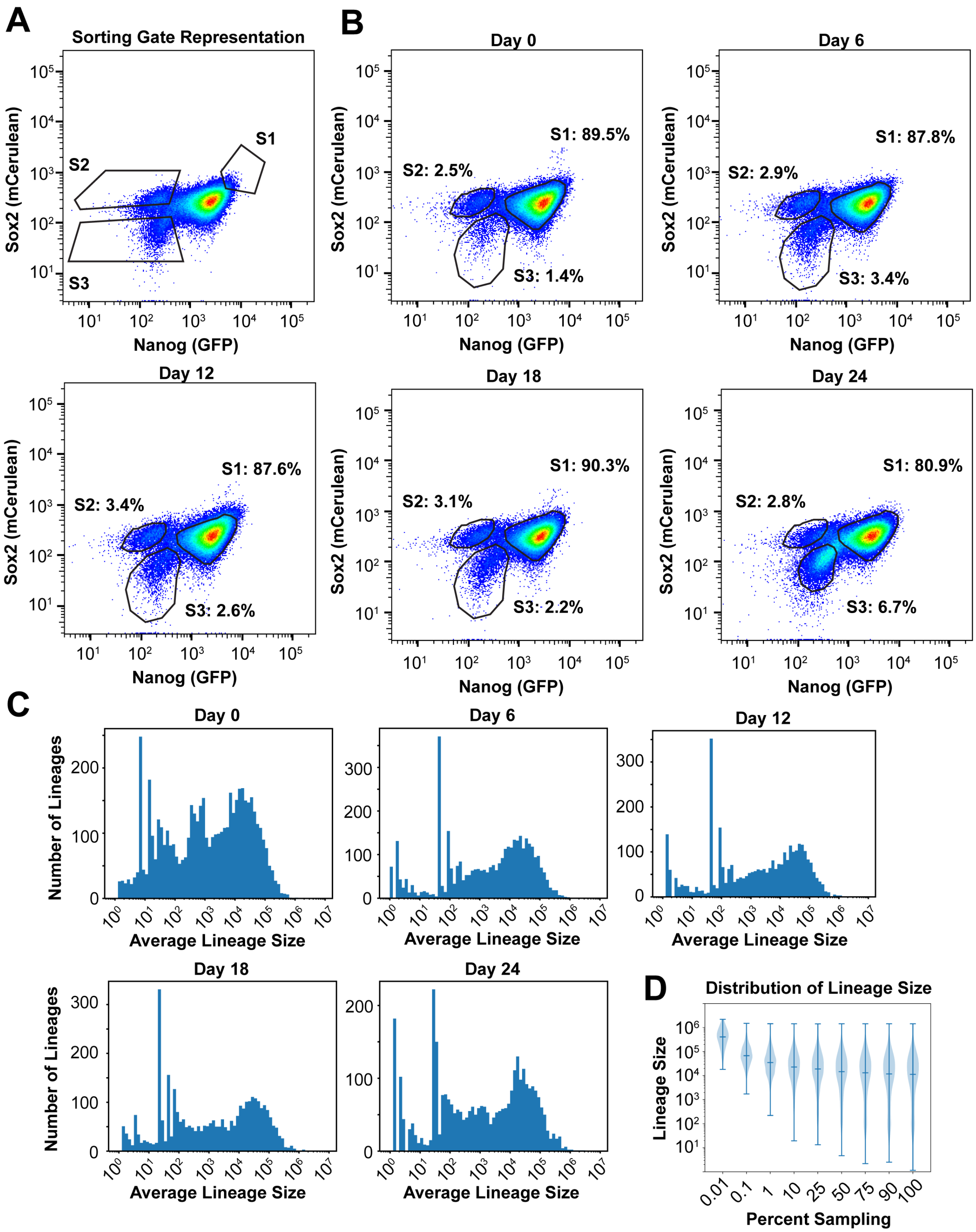

A
Sorting Gate Representation
Sox2 (mCerulean)
Nanog (GFP)
S1
S2
S3
B
Day 0
S1: 89.5%
S2: 2.5%
S3: 1.4%
Day 6
S1: 87.8%
S2: 2.9%
S3: 3.4%
Day 12
S1: 87.6%
S2: 3.4%
S3: 2.6%
Day 18
S1: 90.3%
S2: 3.1%
S3: 2.2%
Day 24
S1: 80.9%
S2: 2.8%
S3: 6.7%
C
Number of Lineages
Average Lineage Size
D
Distribution of Lineage Size
Lineage Size
Percent Sampling
0.01
0.1
1
10
25
50
75
90
100

Figure S2. ESC lineage state composition over time, related to Figure 1. (A) Schematic showing representative gates used to sort populations for States 1-3 at each timepoint of the experiment. Sorted cells were analyzed for >99% purity from the other two gates. Note extreme gates are chosen to ensure purity of the sorted population, and these gates differ from those that are used to consider the portion of cells contained within each of the three states. **(B)** The proportion of ESC in States 1-3 at each timepoint of the experiment is shown, along with the static gates used to determine state membership. **(C)** Distribution of ESC lineage sizes at each timepoint of the experiment. **(D)** Distribution of lineage sizes as data (reads) are sub-sampled. Percent sampling refers to the proportion of randomly selected reads from the FASTQ file that were retained for analysis, and the distribution across inferred lineage sizes is shown. The median (center line) and 1st-99th percentiles (whiskers) are indicated.

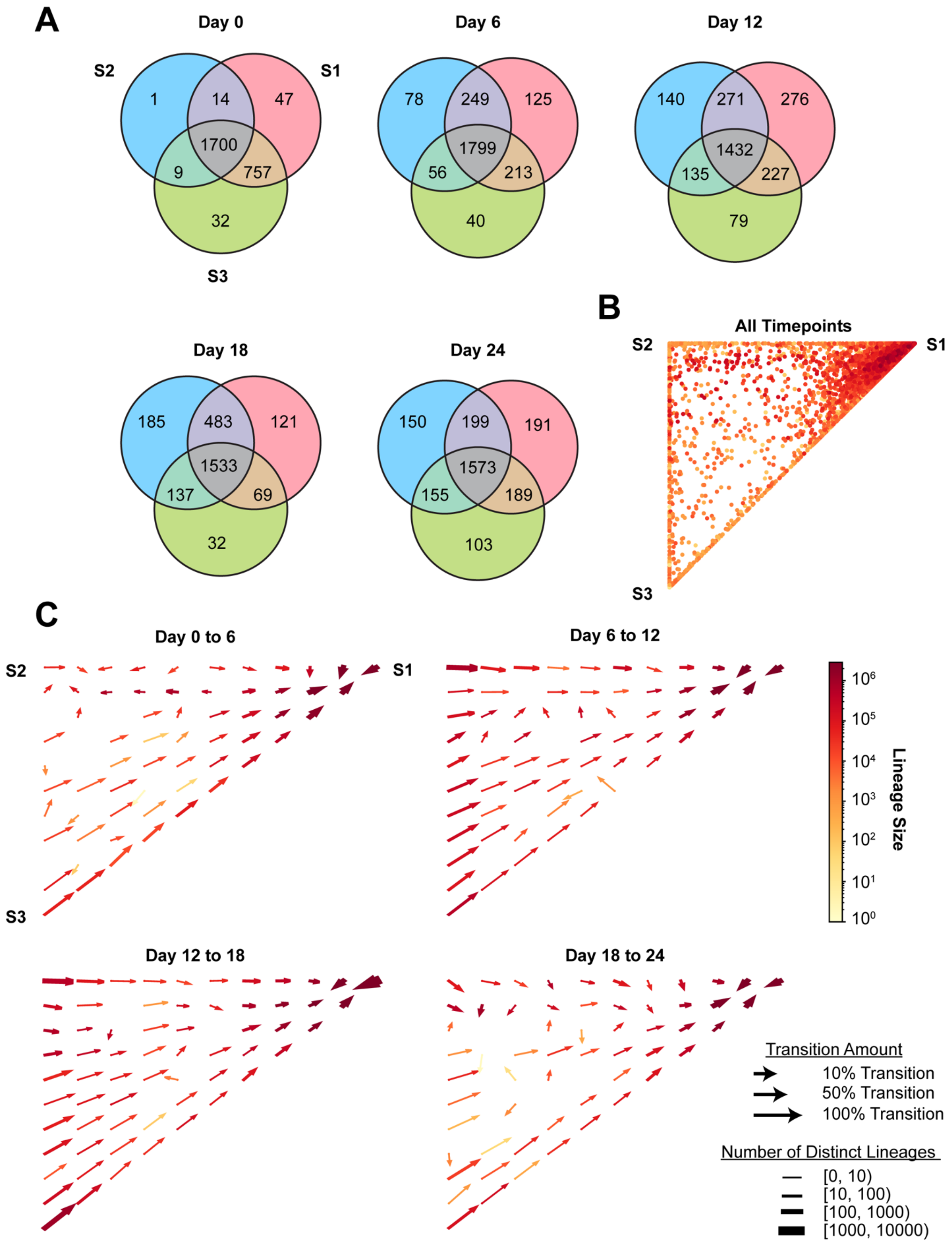
A
Day 0
S2
S1
S3
1
14
47
1700
9
757
32
Day 6
78
249
125
1799
56
213
40
Day 12
140
271
276
1432
135
227
79
Day 18
185
483
121
1533
137
69
32
Day 24
150
199
191
1573
155
189
103
B
All Timepoints
S2
S1
S3
C
Day 0 to 6
S2
S1
S3
Day 6 to 12
Day 12 to 18
Day 18 to 24
Lineage Size
10^0
10^1
10^2
10^3
10^4
10^5
10^6
Transition Amount
10% Transition
50% Transition
100% Transition
Number of Distinct Lineages
[0, 10)
[10, 100)
[100, 1000)
[1000, 10000)

Figure S3. Number and transitions of lineages between states, related to Figure 3. (A) Venn-diagrams showing number of lineages that were detected in each state or in multiple states at all timepoints. **(B)** Ternary dot plot showing the distribution of lineages across three states for all lineages at all timepoints combined. **(C)** Vector field plots showing the local average change in state proportions (state transitions) for each of the four measured transitions between timepoints in the experiment. Color bar for panels B, C indicate lineage size in number of cells.

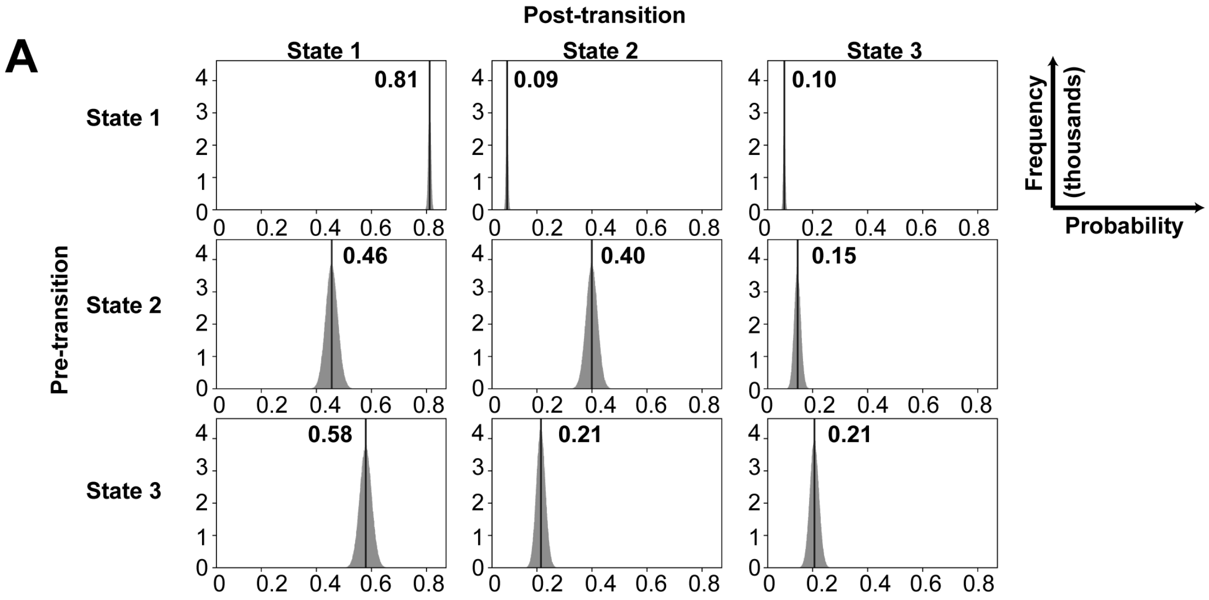

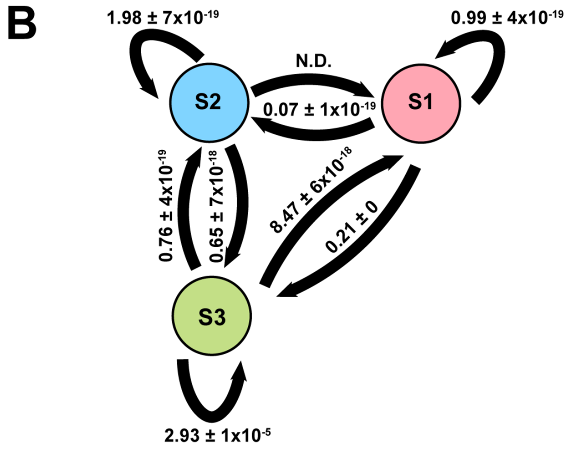

Transitions	Mean	95% CI
S1 to S1	0.99	[0.99 0.99]
S1 to S2	0.07	[0.07 0.07]
S1 to S3	0.21	[0.21 0.21]
S2 to S1	-1.7	[-1.7 -1.7]
S2 to S2	1.98	[1.98 1.98]
S2 to S3	0.65	[0.65 0.65]
S3 to S1	8.47	[8.47 8.47]
S3 to S2	0.76	[0.76 0.76]
S3 to S3	2.93	[2.93 2.93]

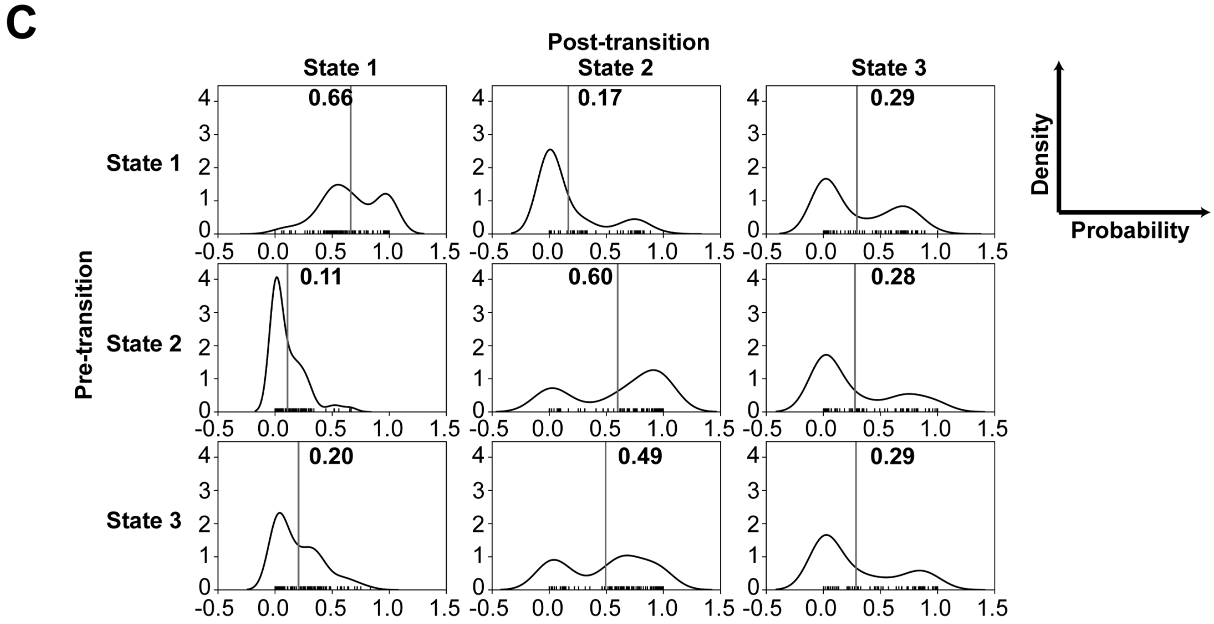

Figure S4. Confidence interval estimation for matrices used in Markov models, related to STAR Methods. (A) We estimated confidence intervals for the parameters of the Markov model (Fig. 4A) by bootstrapping 80% of lineages (out of 2,560) over 10^5 iterations. The distribution for each parameter in the transition rate across these iterations is shown. **(B)** (left) Growth-Birth-Death rates for cell populations making each kind of transition (see Note). For example, cells remaining in State 2 show a net growth-birth-death rate of 1.98, meaning such cells nearly double in size between timepoints. Pink circle, Blue circle and Green circle represent State 1, State 2 and State 3, respectively. Rate estimates are given +/- standard error of the mean calculated using 80% bootstrapping over 10^5 iterations. (right) 95% confidence intervals for parameters, estimated by bootstrapping, are given. **(C)** Distribution of Markov transition matrix parameters for the 114 lineages most out of equilibrium (non-Markov transitions across all timepoints) shown as kernel density plots (line) and rug plots (underneath). Mean values are indicated.

Number of shared non-Markovian motifs between different thresholds

	(0, 50, 50)	(10, 45, 45)	(20, 40, 40)	(30, 35, 35)	(40, 30, 30)	(50, 25, 25)	(60, 20, 20)	(70, 15, 15)	(80, 10, 10)	(90, 5, 5)	Plurality vote
(0, 50, 50)	16	16	16	16	14	14	13	11	8	7	13
(10, 45, 45)		16	16	16	14	14	13	11	8	7	13
(20, 40, 40)			16	16	14	14	13	11	8	7	13
(30, 35, 35)				16	14	14	13	11	8	7	13
(40, 30, 30)					14	14	13	11	8	6	13
(50, 25, 25)						17	14	12	9	7	14
(60, 20, 20)							18	15	12	10	14
(70, 15, 15)								23	15	13	11
(80, 10, 10)									25	17	8
(90, 5, 5)										29	6
Plurality vote											14

(S1, S2, S3) = minimum percentage of State 1, State 2, and State 3 for a lineage to be assigned to a specific state

Figure S5. Evaluation of plurality vote threshold for establishing lineage state, related to STAR Methods. (top) State membership was evaluated using different thresholds for the fraction of cells from each lineage in States 1-3, given as (S1, S2, S3) thresholds. Plurality vote represents (33.3, 33.3, 33.3). 14 different lineage state sequence motifs are part of non-Markovian pairs in their transition between days 18 -> 24 (see Supplemental Table 2) by plurality vote. The number of lineage state sequence motifs that are part of non-Markov pairs for other thresholds are shown (diagonal), as are the number of these motifs shared between different thresholds. The relatively large number of shared sequence motifs suggests changing thresholds for state membership of a lineage would not alter the qualitative result of some non-Markov dynamics.

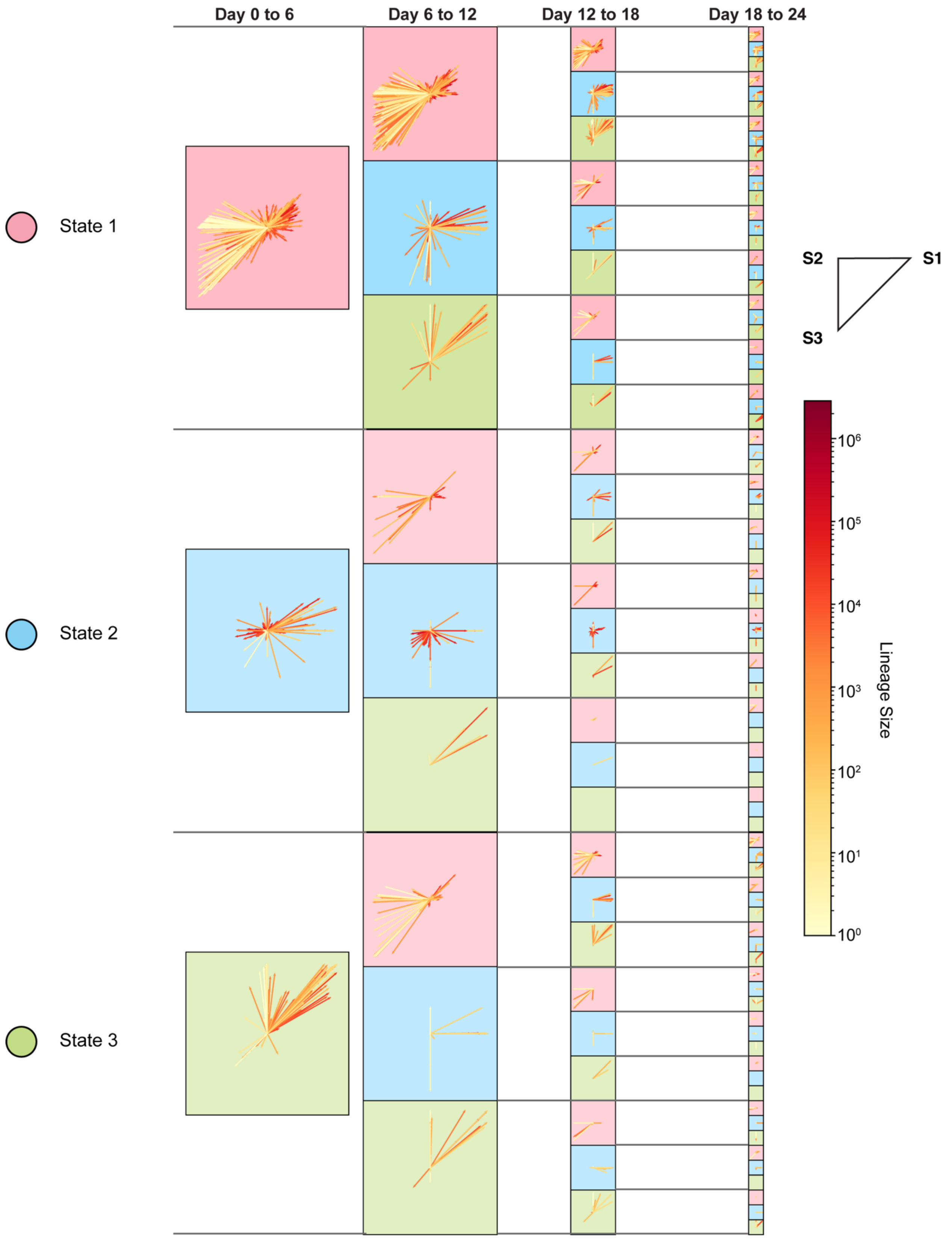

Day 0 to 6
Day 6 to 12
Day 12 to 18
Day 18 to 24
State 1
State 2
State 3
S2
S1
S3
10^6
10^5
10^4
10^3
10^2
10^1
10^0
Lineage Size

Figure S6. Recenter plots for lineages in States 1-3 transitioning over the course of the experiment, related to Figure 4. Recenter plots showing how lineages in different states transition over the four measured transitions from Day 0 to Day 24. Each lineage is assigned a state (S1, S2, S3) on Day 0 based on where a majority of its constituent cells are detected, and is represented by a vector. Each plot follows a collection of transition vectors from lineages in a particular state that follow a particular set of transitions across time. These vectors are then recentered at the origin point (0,0) of the group of vectors. Vector direction indicates the transition of each lineage with respect to a ternary plot (shown at left) across the indicated timepoints. For example, a lineage in S1 at day 0 represented here by a left arrow vector (-1,0) indicates this lineage transitions entirely to S2, and a lineage represented by vector (-0.5,-0.5) would indicate an S1 lineage transitioning equally to States 2 and 3. The length of each vector correlates with the change in lineage size, and coloration indicates the present size. Color scale matches that used in main Figure 3 & 4.

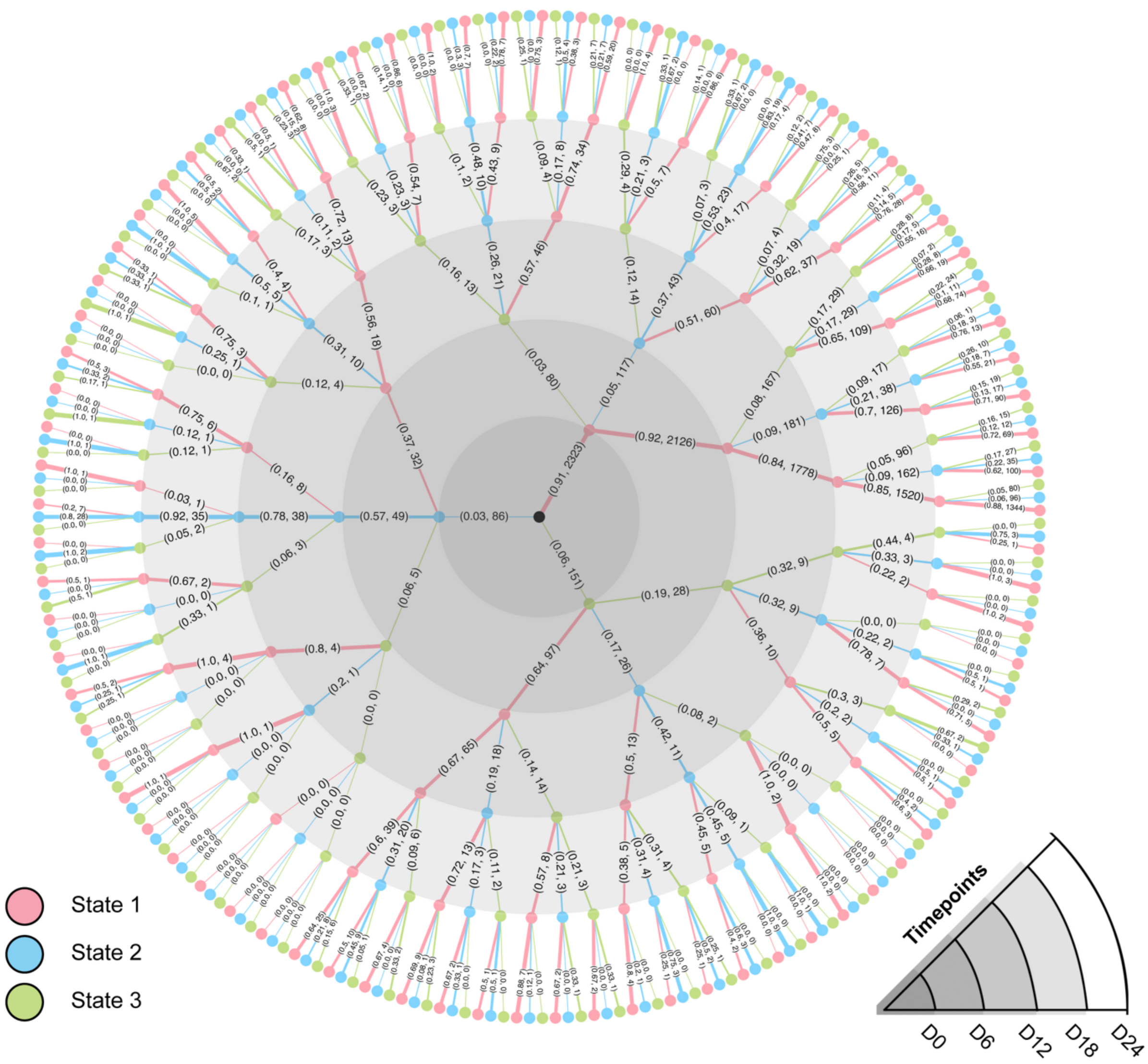

State 1
State 2
State 3
Timepoints
D0
D6
D12
D18
D24

Figure S7. Probability decision tree of lineage Nanog-Sox2 state transitions, related to Figure 4 and STAR Methods. Diagram showing the likelihood of state transitions of ESC lineages in particular Nanog-Sox2 states at all timepoints. Day 0 is represented as the innermost dot, moving outward to Days 6, 12, 18, and 24. Pink, blue, and green dots represent States 1, 2, and 3, respectively. Numbers represent the conditional probability of transition and the number of lineages making said transition down that branch as a (x,y) pair. Thickness of edge connections between transitions are weighted by the conditional probability of transition.

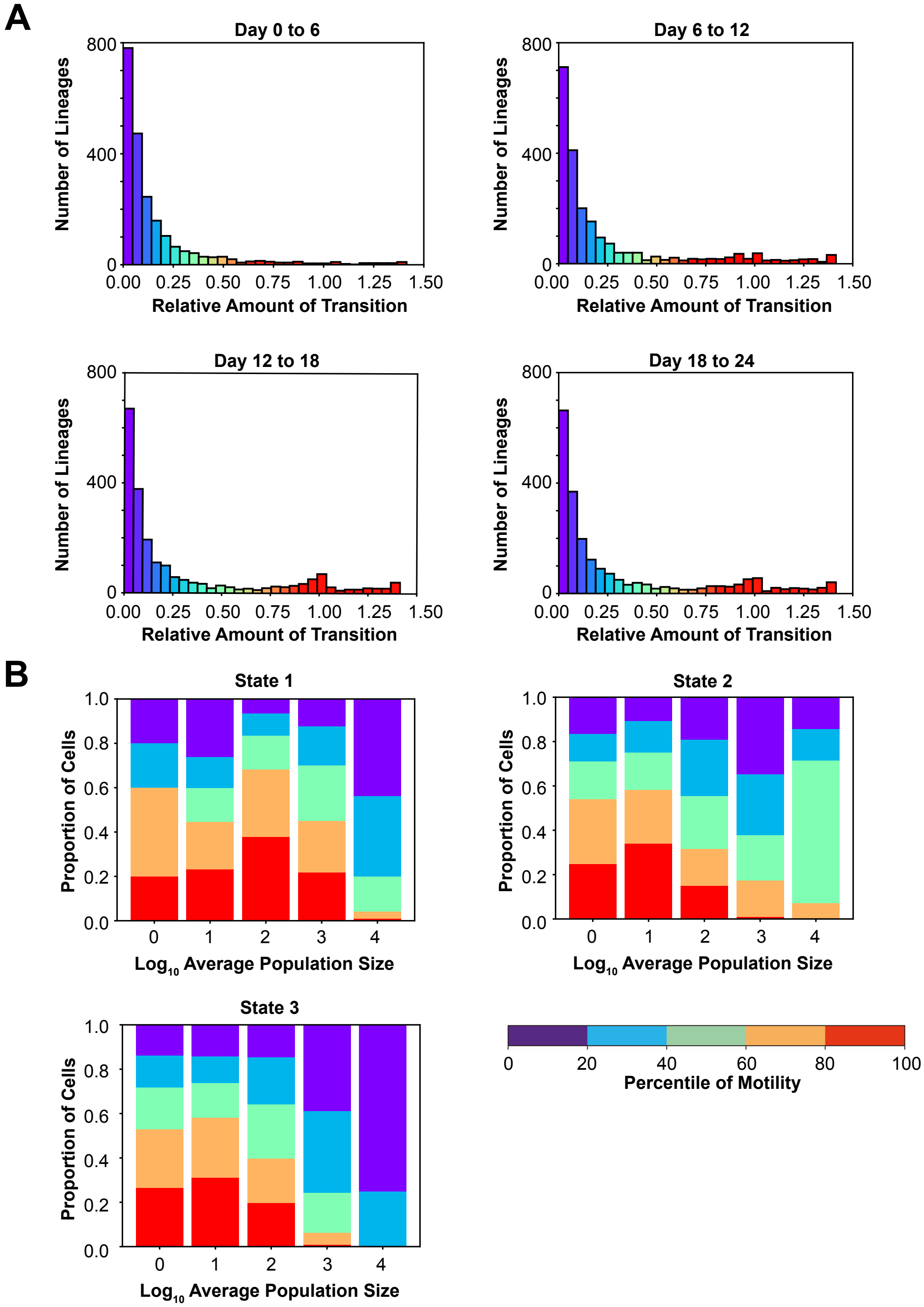
A
Day 0 to 6
Day 6 to 12
Day 12 to 18
Day 18 to 24
Number of Lineages
Relative Amount of Transition
800
400
0
0.00
0.25
0.50
0.75
1.00
1.25
1.50
B
State 1
State 2
State 3
Proportion of Cells
Log10 Average Population Size
1.0
0.8
0.6
0.4
0.2
0.0
0
1
2
3
4
Percentile of Motility
0
20
40
60
80
100

Figure S8. Motility analysis of ESC lineages, related to Figure 5 and STAR Methods. (A) Histograms showing the distribution of lineages with different amount of transition between states (motility) at each transition point. Lineages are colored as in main Figure 5. **(B)** Stacked bar plots revealing the proportion of lineages of different sizes (binned by order of magnitude) with different motilities in each state, summed across all four transitions. The state of a lineage is defined as where a majority of its ESCs are located at the first timepoint of the transition. Lineages are grouped into 5 quintiles of transition: red (top 80-100th percentiles), orange (60-80th percentiles), teal (40th-60th percentiles), blue (20th-40th percentiles), violet (0th-20th percentiles). Note the highest transitioning lineages (red) are not only present in smallest size bin.

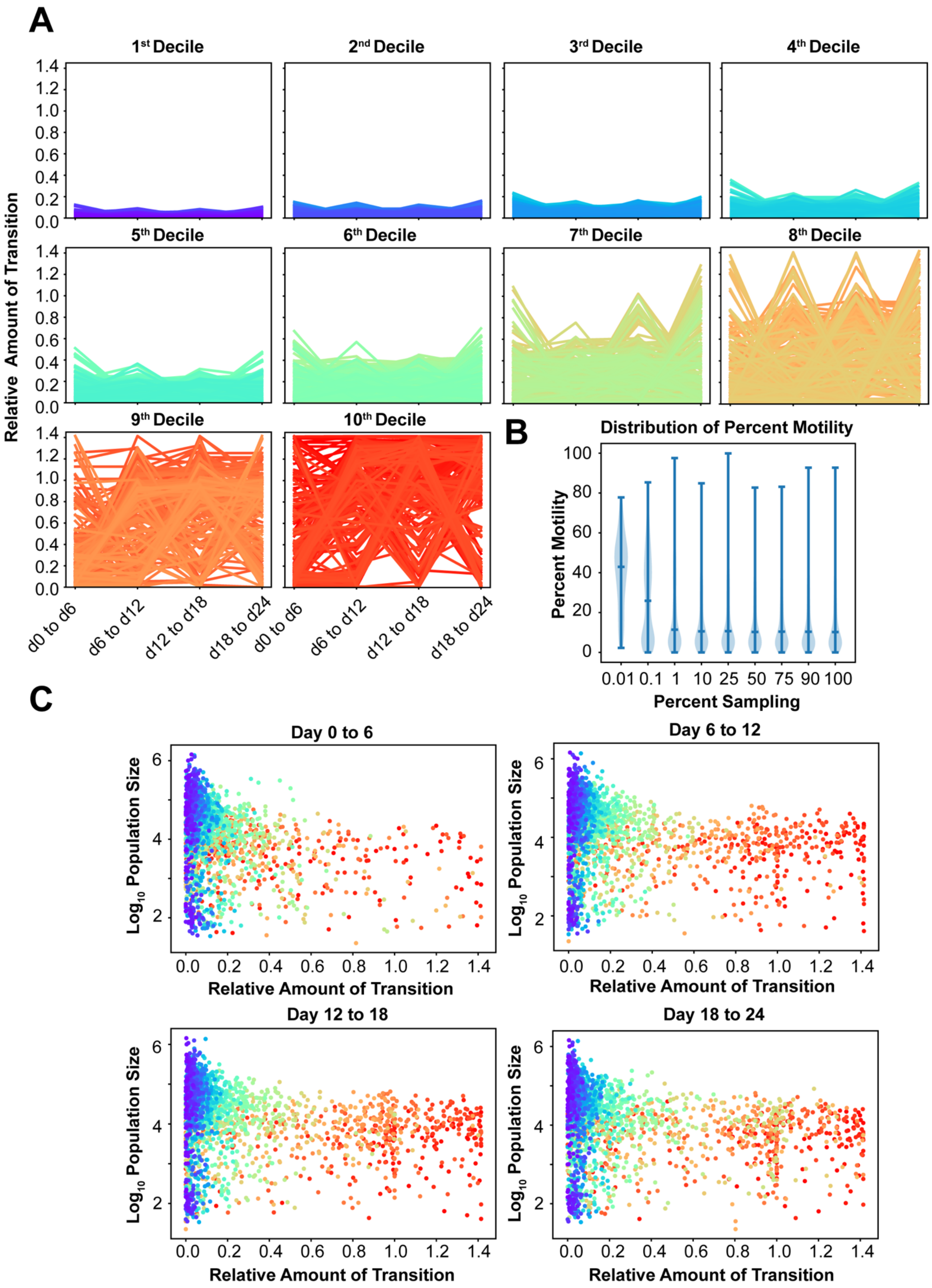
A
1st Decile
2nd Decile
3rd Decile
4th Decile
5th Decile
6th Decile
7th Decile
8th Decile
9th Decile
10th Decile
Relative Amount of Transition
d0 to d6
d6 to d12
d12 to d18
d18 to d24
B
Distribution of Percent Motility
Percent Motility
Percent Sampling
0.01 0.1 1 10 25 50 75 90 100
C
Day 0 to 6
Day 6 to 12
Day 12 to 18
Day 18 to 24
Log10 Population Size
Relative Amount of Transition

Figure S9. Decile motility plots and relationship between motility and lineage size, related to Figure 5 and STAR Methods. **(A)** Line charts showing the amount of transition for each lineage by decile of overall motility. 1st and 10th deciles are also shown in main Figure 5C. **(B)** Violin plot showing the distribution of lineage motility as data (reads) are sub-sampled. Percent sampling refers to the proportion of randomly selected reads from the FASTQ file that were retained for analysis, and the distribution of motility (percent transition) is shown. The median (center line) and 1st-99th percentiles (whiskers) are indicated. **(C)** Scatter plots showing the amount of transition and sizes of each lineage in each transition. Each dot is colored by the overall amount of that lineage's transition across all timepoints – colors match those used in Figure 5.

A

	Day 0 to 6	Day 6 to 12	Day 12 to 18	Day 18 to 24
**Day 0 to 6**	1.000	0.386	0.190	0.209
**Day 6 to 12**		1.000	0.500	0.255
**Day 12 to 18**			1.000	0.420
**Day 18 to 24**				1.000

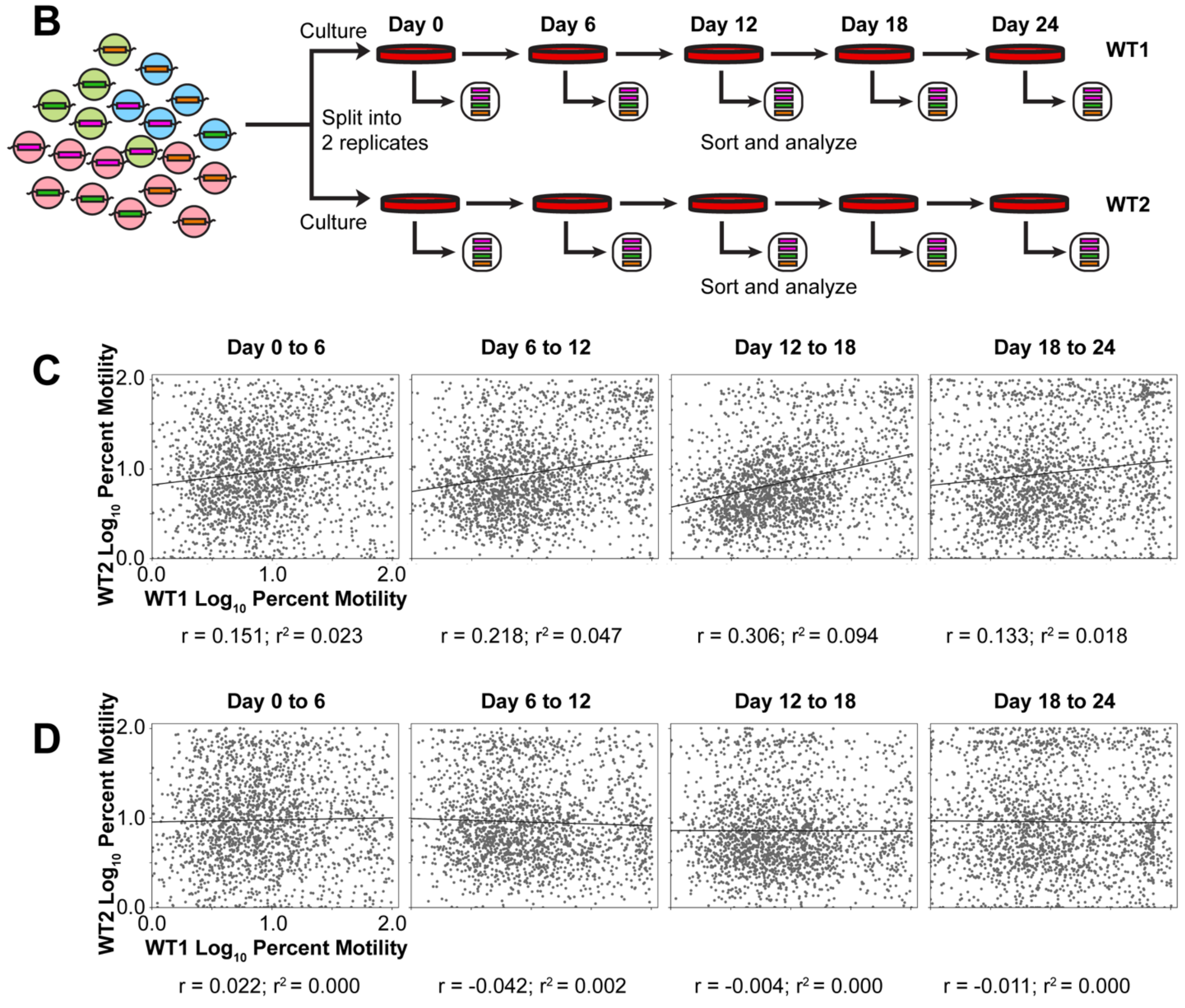

Figure S10. Correlation between replicates, related to STAR Methods. (A) Matrix showing the Pearson correlation (r-value) for motility of all lineages when different transitions are plotted against one another. **(B)** Schematic of replicate experiment. The initial barcoded population of ESC lineages was split into two equal portions. Each was maintained separately over 24 days, and ESC lineages in States 1-3 were assessed by sorting representative pools from States 1-3 on Days 0, 6, 12, 18, and 24. Barcodes were sequenced and the relative proportion of each lineage in each state assessed. **(C)** Scatter plots depicting the relationship between percent transition amounts in replicate 1 (WT1) and replicate 2 (WT2) at all transitions in the experiment. The Pearson correlation (r-value) and r^2 are shown. **(D)** Scatter plots depicting the relationship between percent transition amounts in randomized replicates. In each replicate, transition amounts were chosen randomly for each lineage from the empirical distribution of transition amounts and then plotted against each other. No significant correlation is observed.

A

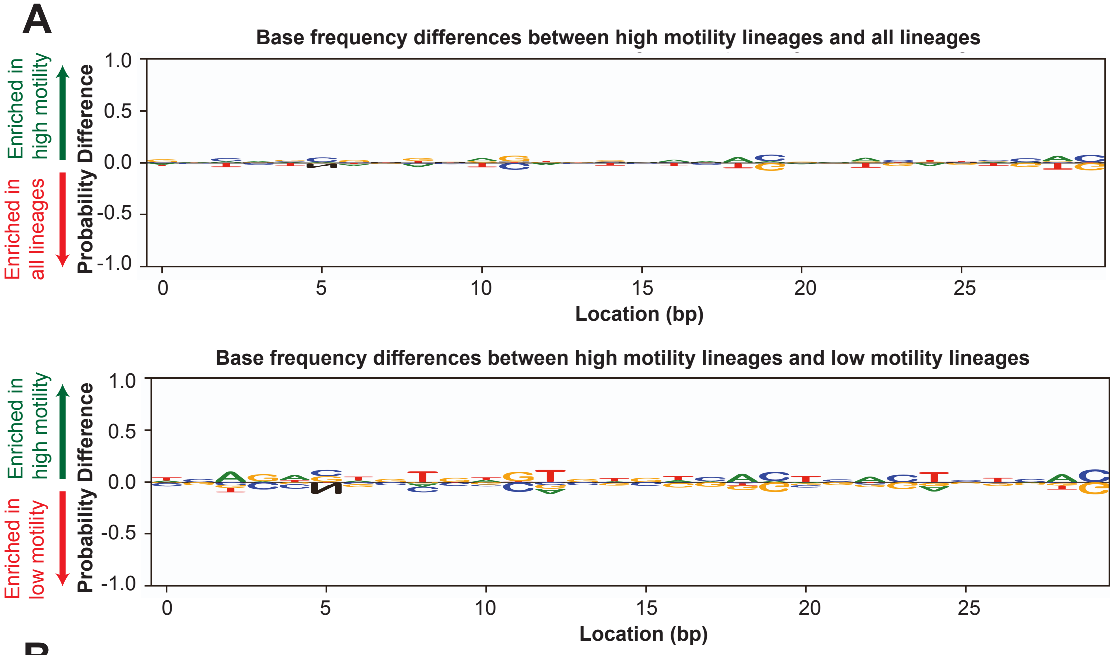

B

	Day 0 to 6	Day 6 to 12	Day 12 to 18	Day 18 to 24
**Day 0 to 6**	1.000	0.184	0.001	-0.002
**Day 6 to 12**		1.000	0.204	-0.003
**Day 12 to 18**			1.000	0.142
**Day 18 to 24**				1.000

Figure S11. Amplification bias cannot account for motility correlation across distal transitions, related to Figure 5. (A) The base frequency probability across high motility lineages (top decile) as compared to all lineages and as compared to low motility lineages (bottom decile) is shown across the 30bp barcoding region. No significant base differences are noted. **(B)** To simulate random PCR amplification, read counts were randomly reassigned to lineages within each sample (particular state on a particular day), motility for each lineage calculated, and compared across transitions. Note there is no motility correlation between the first and third or fourth transitions under random amplification. Weak correlation is observed under random reassignment between adjacent transitions because lineages assigned read counts across states distributed away from the equilibrium point are more likely to randomly be assigned to the equilibrium at the next time point (since most data points are at equilibrium), resulting in some apparent motility. The magnitude of this effect is weaker than the empirically observed motility correlation between adjacent transitions in Fig. S9A.

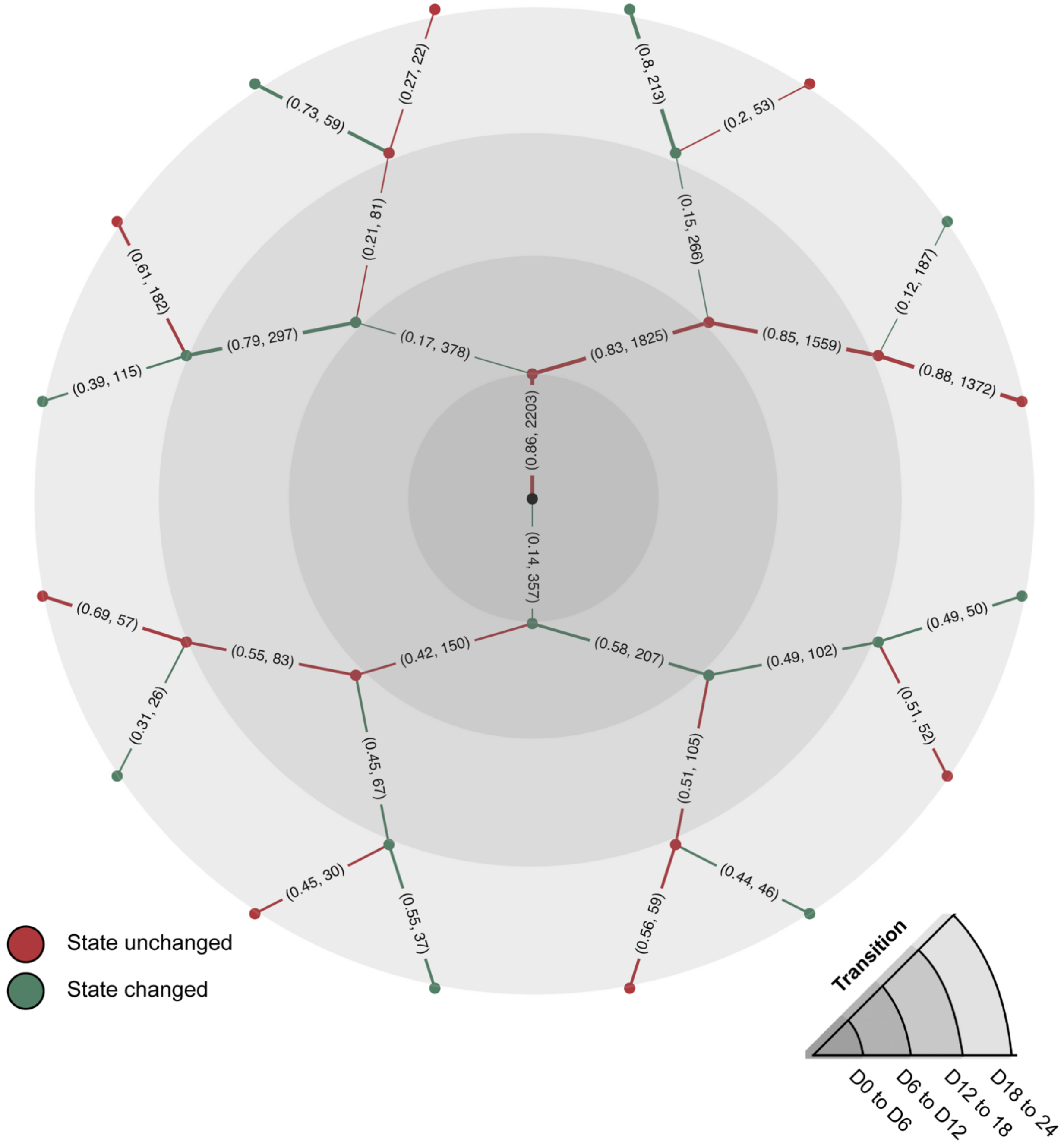
(0.86, 2203)
(0.14, 357)
(0.17, 378)
(0.83, 1825)
(0.42, 150)
(0.58, 207)
(0.79, 297)
(0.21, 81)
(0.15, 266)
(0.85, 1559)
(0.55, 83)
(0.45, 67)
(0.51, 105)
(0.49, 102)
(0.73, 59)
(0.27, 22)
(0.8, 213)
(0.2, 53)
(0.61, 182)
(0.39, 115)
(0.12, 187)
(0.88, 1372)
(0.69, 57)
(0.31, 26)
(0.45, 30)
(0.55, 37)
(0.56, 59)
(0.44, 46)
(0.49, 50)
(0.51, 52)
State unchanged
State changed
Transition
D0 to D6
D6 to D12
D12 to 18
D18 to 24

Figure S12. Probability decision tree of lineage motility state transitions, related to Figure 5. Diagram showing the likelihood of state transitions of ESC lineages in particular motility states at all timepoints. Day 0 is represented as the innermost dot, moving outward to Days 6, 12, 18, and 24. Red and green dots represent lowly motile and highly motile states, respectively. Numbers represent the conditional probability of transition and the number of lineages making said transition down that branch as a (x,y) pair. Thickness of edge connections between transitions are weighted by the conditional probability of transition.

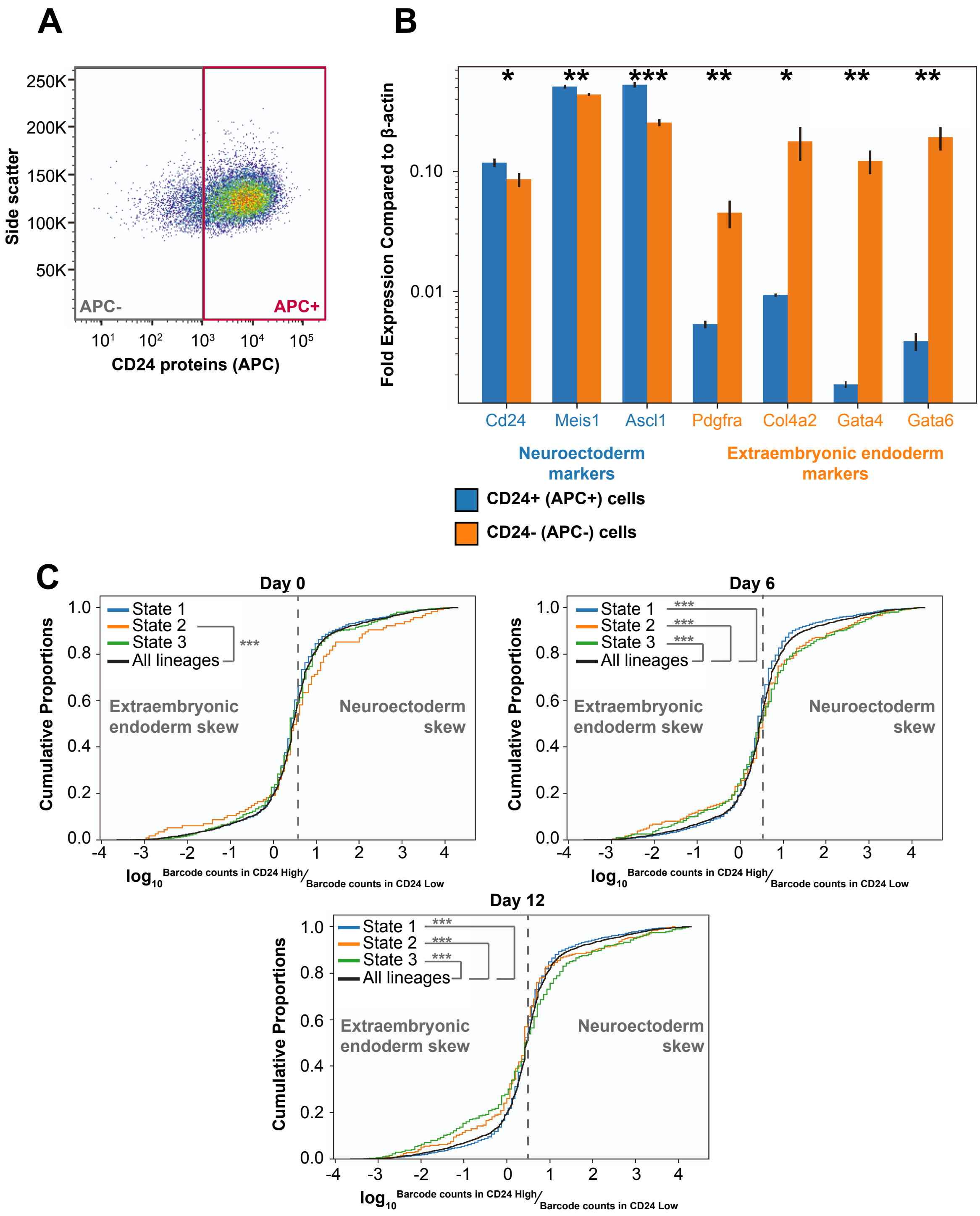

A
Side scatter
250K
200K
150K
100K
50K
APC-
APC+
CD24 proteins (APC)
B
Fold Expression Compared to β-actin
0.10
0.01
Cd24
Meis1
Ascl1
Pdgfra
Col4a2
Gata4
Gata6
Neuroectoderm markers
Extraembryonic endoderm markers
CD24+ (APC+) cells
CD24- (APC-) cells
C
Day 0
Day 6
Day 12
State 1
State 2
State 3
All lineages
Cumulative Proportions
Extraembryonic endoderm skew
Neuroectoderm skew
log10 Barcode counts in CD24 High/Barcode counts in CD24 Low

Figure S13. Differentiation of ESC lineages in retinoic acid, related to Figure 6 and STAR Methods. (A) Sorting gate used to separate CD24^{high} (neuroectoderm) and CD24^{low} (extraembryonic endoderm) cells. **(B)** RT-qPCR for markers of neuroectoderm and extraembryonic endoderm. Measurements were performed in triplicate and are representative of two experiments. P-values for two-sample t-tests are shown. Asterisks indicate levels of significance (* $p < 0.05$, ** $p < 0.01$, *** $p < 0.001$). **(C)** For lineages in States 1-3 at day 0, the ratio of descendants in CD24^{high} vs CD24^{low} populations is plotted. Similar plots are shown for lineage state at day 6 and day 12. F-tests for variance were conducted comparing each state to all lineages and p-values < 0.001 (***) are indicated.

Cell numbers and timeline for main experiment

Timepoints	Description
Day -14	Plate cells for viral transduction
Day -13	Transduce with lentivirus encoding genomic barcode at low MOI
Day -9	Isolate 101,703 cells by flow cytometric sorting for RFP+
Day -9 to Day 0	Expand 101,703 cells by growth, make freezes of transduced library at Days -5, -3, -1
Day 0	Begin sorting experiment below, 10^6 cell aliquot taken to assess barcode representation
Day 0	5,431 unique barcodes are found represented in the above pool of 10^6 cells

	Isolated Cell Numbers		
**Timepoints**	**State 1**	**State 2**	**State 3**
Day 0	272747	286525	432122
Day 6	333166	308834	333166
Day 12	355867	366254	400000
Day 18	338623	400000	357263
Day 24	349522	349695	400000

Table S1. Cell numbers and timeline of the main experiment, related to Figure 1.

File: 190812Gar.fastq	
Categories	Number
Total number of Reads	374225752
Total number of Reads that passed the quality metrics	221544591
Total number of Reads with bad barcoding region	68461911
Total number of Reads with bad constant regions	82732636
Total number of Reads with bad sample index	1486614
File: 191003Gar.fastq	
Categories	Number
Total number of Reads	14665372
Total number of Reads that passed the quality metrics	11396578
Total number of Reads with bad barcoding region	333848
Total number of Reads with bad constant regions	2910948
Total number of Reads with bad sample index	23998
File: 200623Gar.fastq	
Categories	Number
Total number of Reads	340613796
Total number of Reads that passed the quality metrics	198785419
Total number of Reads with bad barcoding region	32014785
Total number of Reads with bad constant regions	109026377
Total number of Reads with bad sample index	787215
File: 200707Gar.fastq	
Categories	Number
Total number of Reads	192061270
Total number of Reads that passed the quality metrics	115409800
Total number of Reads with bad barcoding region	6068656
Total number of Reads with bad constant regions	69975250
Total number of Reads with bad sample index	607564

Table S4. Summary of the number of reads before and after the quality control, related to STAR Methods.

Genes	Primers	Sequence (5' -> 3')
Hoxb1	Forward	GCCTACGACCTCCTCTCTGA
	Reverse	CGGACACCTTCGCTGTCTT
Meis1	Forward	CACACTGCTGGAGACGCAAA
	Reverse	GGGGTAGGTCGTCGTACCTTT
Ascl1	Forward	CTCGTCCTACTCCTCCGACG
	Reverse	GATCTGCTGCCATCCTGCTTC
Pdgfra	Forward	GGAACCTCAGAGAGAATCGGC
	Reverse	CATAGCTCCTGAGACCCGCT
Cd24a	Forward	GCTCCTACCCACGCAGATTT
	Reverse	GACGTTTCCTGGCCTGAGTC
Col4a2	Forward	CGTGACAAATACCGGGGTGA
	Reverse	GTCCGTAAAAACCGAGCCCT
Gata4	Forward	AATCTAAGACGCCAGCAGGTC
	Reverse	CGGACACAGTACTGAATGTCTGG
Gata6	Forward	CAGTTTTCCGGCAGAGCAGT
	Reverse	TCCAACCTGACTTTTGATTCCTCG

Table S6. RT-qPCR primers used in quantifying markers of neuroectoderm and extraembryonic endoderm lineages, related to STAR Methods.
